# *Ducrosia* spp., Rare Plants with Promising Phytochemical and Pharmacological Characteristics: An Updated Review

**DOI:** 10.3390/ph13080175

**Published:** 2020-07-31

**Authors:** Javad Mottaghipisheh, Anahita Boveiri Dehsheikh, Mohammad Mahmoodi Sourestani, Tivadar Kiss, Judit Hohmann, Dezső Csupor

**Affiliations:** 1 Department of Pharmacognosy, Faculty of Pharmacy, University of Szeged, Eötvös u. 6, 6720 Szeged, Hungary; kiss.tivadar@pharmacognosy.hu (T.K.); hohmann@pharm.u-szeged.hu (J.H.); 2 Department of Horticultural Science, Faculty of Agriculture, Shahid Chamran University of Ahvaz, Ahvaz 61357-43311, Iran; anahitaboveiri84@gmail.com (A.B.D.); m.mahmoodi@scu.ac.ir (M.M.S.); 3 Interdisciplinary Centre for Natural Products, University of Szeged, Eötvös u. 6, 6720 Szeged, Hungary

**Keywords:** *Ducrosia* genus, folk medicine, phytoconstituents, non-volatile compounds, VOC, bioactivity

## Abstract

The rare genus *Ducrosia* (Apiaceae family) consists of six species, which are mainly native to Asia, specifically to Iran and Iraq. The aerial parts of *D. anethifolia,* as the most common species, have been traditionally consumed to relieve headache, backache and colic pain, and have also been used as an anxiolytic, an antidepressant, and for treating insomnia. The antispasmodic and carminative effects of *D. assadii*, and the analgesic activity of *D. flabellifolia*, along with the insecticidal activities and use as a remedy of skin infections of *D. ismaelis*, have been previously documented. Among the 49 non-volatile secondary metabolites identified from *D. anethifolia* and *D. ismaelis*, 17 linear furanocoumarins and 8 flavonoids have been characterized. The essential oil compositions of four species, including *D. anethifolia*, *D. assadii*, *D. flabellifolia* and *D. ismaelis*, have been analyzed, whereby aldehyde hydrocarbons, including decanal (10.1–74.0%) and dodecanal (7.2–33.41%), and α-pinene (4.0–70.3%), were identified as the main aroma constituents. From the species of the genus, the bioactivities of *D. anethifolia*, as well as *D. ismaelis*, *D. assadii* and *D. flabellifolia*, have been previously investigated. Except one clinical trial, all the pharmacological data are derived from preclinical tests, predominantly focusing on antimicrobial, antioxidant, antiproliferative and cytotoxic activities in vitro, and neuroprotective, antidiabetic and analgesic effects in vivo. Considering the vast ethnobotanical uses of the plants in Iranian folk medicine, the phytochemical and pharmacological analysis of un-investigated species might be promising. Furthermore, due to extensive consumption of the *Ducrosia* genus, more scientific data are needed to support the safety and efficacy of these plants.

## 1. Introduction

The Apiaceae (syn. Umbelliferae) family, comprising 446 genera and 3820 species, is one of the largest plant families. The members of this family are mostly aromatic plants with hollow stems. The family is famed as the parsley, celery or carrot family. Most of the species have been used in cuisine as condiments and vegetables, and some of them have been consumed for medicinal purposes [1,2,3].

The genus *Ducrosia* (Apiaceae) consists of six species: *D. ismaelis* Asch., *D. flabellifolia* Boiss., *D. assadii* Alava., *D. areysiana* (Deflers) Pimenov & Kljuykov, *D. inaccessa* (C.C.Towns.) Pimenov & Kljuykov and *D. anethifolia* (DC.) Boiss., which are mainly distributed in Africa and Asia, specifically in Iran, Afghanistan, Pakistan, Syria, Lebanon and Iraq [4,5,6,7].

*D. anethifolia*, as the most common plant in the genus and with local names “Moshgak”, “Roshgak” and “Moskbu” in Iran [8], is an herbaceous and biennial plant with a height of 10–30 cm. The stems are glabrous and branched with ramified and ovate-oblong leaves, with white florets in umbel inflorescence [9].

*D. flabellifolia,* possessing three lobed leaves with cuneate, flabelliform and flat segments, and yellow flowers [6] with the local name of “Al Haza”, grows as a rare species in volcanic cinders in the middle and north of Saudi Arabia [10,11,12], and in the deserts of eastern parts of Jordan [13].

*D. assadii* is an herbaceous and perennial plant (height 25–66 cm). The species has branched leaves and an umbel inflorescence with white flowers [14].

The documented ethnomedicinal application of *Ducrosia* species is limited to four species: *D. anethifolia*, *D. assadii*, *D. flabellifolia* and *D. ismaelis* (Table 1). *D. anethifolia* is known as the most popular plant in the genus. The aerial parts of *D. anethifolia* have been traditionally used to relieve headache, backache and colic pain in Asian countries, particularly Afghanistan, Iran, Iraq, Pakistan and Lebanon [4,15,16,17,18,19,20,21]. The effects of the aerial parts of *D. anethifolia* on the central nervous system were also reported, especially its use as anxiolytic, antidepressant and in treating insomnia [17,21,22,23]. For this plant, the following traditional applications have also been documented: treatment of cold [7,17,21,24], heartburn [21] and inflammation of the inner wall of the nose [7], and as an analgesic [4,17,18,20,25], a flavoring in food [4,23,26,27,28,29,30] and an insecticide [7,17,21,24]. The decoction prepared from aerial parts, leaves and seeds of *D. anethifolia* possesses carminative and lactiferous effects [31,32,33,34].

The aerial parts of *D. assadii* have been applied as an analgesic, a remedy for cold, because of their anti-inflammatory, antiseptic and soporific effects, and also as food additives in Iranian folk medicine [14,29,35]. In addition, the antispasmodic and carminative potentials of this species have been described in the Kerman province of Iran [23,26].

From ancient times, the aerial parts and leaves of *D. flabellifolia* have been smoked as a cigarette, since the local people in Iran, Jordan, Saudi Arabia, Iraq and Syria suppose that it can relieve various pains (e.g., backache, headache, toothache) [13,36].

Besides the aforementioned ethnomedicinal uses of the *Ducrosia* genus, the aerial parts of *D. ismaelis* have been used to treat skin infections, and as natural insecticides in some African and Asian countries [2,4,23,24,37].

The majority of pharmacological studies have been carried out with *D. anethifolia*. Except one clinical study, all the experiments have been carried out in preclinical (in vitro and in vivo) settings. Different plant products (isolated compounds, extracts and essential oils (EOs)) have been subjected to bioassays. The in vitro antimicrobial, antioxidant, antiproliferative and cytotoxic activities, along with the in vivo analgesic, anti-inflammatory and neuroprotective effects, are the most thoroughly evaluated bioactivities.

Linear furanocoumarins have been previously identified as the major non-volatile components of the *Ducrosia* genus [2], whilst the main most volatile constituents were aliphatic hydrocarbons, with decanal (10.1–74.0%) [8,13,14,18,19,26,38,39,40,41,42,43,44,45,46,47] and dodecanal (7.2–33.41%) as the major constituents [8,13,14,19,26,39,41,42,43,45,46].

Considering the extensive use of *Ducrosia* species in traditional medicine, the number of studies dealing with this genus is increasing. The present review aims at comprehensively gathering the data on the genus regarding the traditional use, and phytochemical and pharmacological studies, of the *Ducrosia* genus for the first time. Databases including PubMed, Web of Science and SciFinder were used to search the reports using keyword “*Ducrosia*” (last search: 1 May 2020).

## 2. Phytoconstituents

### 2.1. Non-Volatile Components

**Figure 1:**
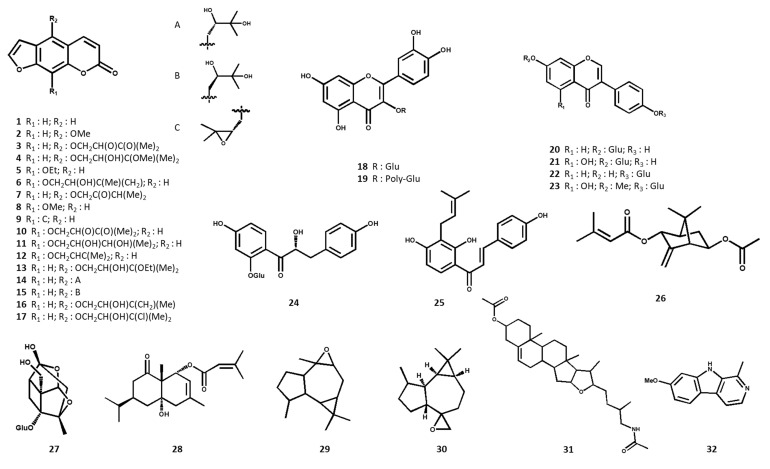

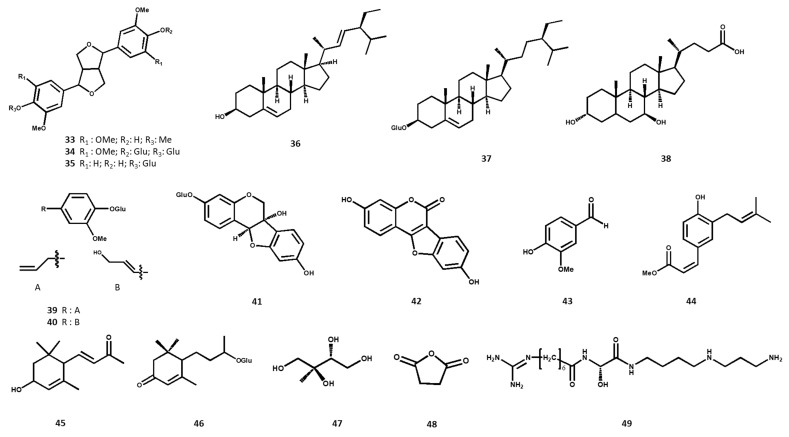
Chemical structures of the non-volatile phytochemicals identified from *Ducrosia* species.

Only two species (*D. anethifolia* and *D. ismaelis*) have been subjected to preparative phytochemical experiments to identify their main non-volatile secondary metabolites. Overall, the characterized compounds comprise 17 linear furanocoumarins (**1**–**17**), 8 flavonoids (**18**–**25**) and 5 terpenoids (**26**–**30**) as the major and characteristic constituents (Table 2). Furanocoumarins, as the major phytoconstituents of the studied species, were most abundant in chloroform extracts of the aerial parts, including stem, leaf and seed. The present flavonoids were identified in hydroalcoholic fractions of *D. ismaelis’* aerial parts. Four compounds have been isolated as new secondary metabolites in the plant kingdom, including one dihydrochalcone glycoside, a new pterocarpan glycoside, one new monoterpene, and a novel sesquiterpene. The chemical structures of the identified non-volatile secondary metabolites are shown in Figure 1.

#### 2.1.1. Furanocoumarins

Coumarin derivatives are considered the most characteristic phytoconstituents of the Apiaceae family [55,56,57]. The aerial parts of *D. anethifolia* collected in Saudi Arabia have been examined so as to isolate and identify its major compounds. Psoralen (**1**) was isolated from the ethyl acetate extract of the leaf and stem of *D. anethifolia*, together with 5-methoxypsoralen (syn. bergapten) (**2**), oxypeucedanin methanolate (**4**), isooxypeucedanin (**7**), 8-methoxypsoralen (syn. xanthotoxin) (**8**), imperatorin (**12**), oxypeucedanin hydrate (**14**) and pabulenol (syn. pangelin) (**16**) [48].

From the ethyl acetate extract of the fresh aerial parts of *D. anethifolia* harvested from Saudi Arabia, 8-ethoxypsoralen (**5**) and prangenin (syn. oxyimperatorin) (**10**) have been identified by GC-MS as the predominant compounds (6.5% and 6.26%, respectively) [49].

In a former study performed by our group, nine linear furanocoumarins were isolated from a chloroform extract obtained from *D. anethifolia*’s aerial parts, harvested from Iran. Oxypeucedanin (**3**), oxypeucedanin methanolate (**4**), isogospherol (**6**), heraclenin (**9**), heraclenol (**11**), imperatorin (**12**) and pabulenol (**16**) were isolated as the major compounds; in addition, by analysis of optical rotations, two isolated diastereomers of (+)-oxypeucedanin hydrate (syn. aviprin) (**14**) and (‒)-oxypeucedanin hydrate (syn. prangol) (**15**) were identified [2]. Moreover, heraclenin (**9**), heraclenol (**11**) and oxypeucedanin hydrate (**14**) have been isolated from a dichloromethane extract of *D. anethifolia* seeds [50].

Stavri et al., (2003) isolated pangelin (syn. pabulenol) (**16**) from the chloroform extract of *D. anethifolia*’s aerial parts, grown in Kuwait [52].

*D. ismaelis* has also been analyzed in order to identify its non-volatile secondary metabolites. Three furanocoumarins, namely oxypeucedanin ethanolate (**13**), oxypeucedanin hydrate (**14**) and saxalin (**17**), were isolated from the chloroform extract of the aerial parts [51].

#### 2.1.2. Flavonoids

Flavonoids have only been identified in *D. ismaelis*. Two glycosylated quercetin derivatives, quercetin-3-glucoside (**18**) and quercetin-3-polyglycoside (**19**), were isolated from the hydroethanolic extracts of the leaves and stems of this species [53].

From the aqueous extract of *D. ismaelis*’ aerial parts, four isoflavone glycosides, including daidzin (**20**), genistin (**21**), daidzein-4′-*O*-β-*D*-glucopyranoside (**22**) and prunetrin (**23**), have been isolated. Moreover, two chalcones, including a new compound ismaeloside A (**25**) and isobavachalcone (**24**), have been isolated [54].

#### 2.1.3. Terpenoids

All the five identified terpenoids in the *Ducrosia* genus have been reportedly extracted from the seeds and aerial parts of *D. anethifolia*. From the dichloromethane extract of the seeds, the new monoterpene and sesquiterpene compounds ducrosin A (**26**) and ducrosin B (**28**) were isolated, respectively [50]. A monoterpene glycoside, 8-*O*-debenzoylpaeoniflorin (**27**), has been isolated from the chloroform extract of the aerial parts [52]. Furthermore, by applying GC-MS, two oxygenated sesquiterpenes, isoaromadendrene epoxide (**29**) and aromadendrene oxide (**30**), have been identified as the major components from the ethyl acetate extract of aerial parts (7.49% and 2.94%, respectively) [49].

#### 2.1.4. Alkaloids

So far, two alkaloids have been identified in the aerial parts of *D. anethifolia*. The GC-MS analysis of the ethyl acetate extract of *D. anethifolia* resulted in the identification of pseudosolasodine diacetate (**31**) (1.5%) [49], whereas its chloroform extract contained a well-known alkaloid, harmine (**32**) [2].

#### 2.1.5. Lignans

By using various chromatographic techniques, two lignan glycosides, along with an aglycon, have been isolated from *D. ismaelis*. The aqueous extract of its aerial parts has allowed the isolation of 4′-hydroxy-3,3′,4,5,5′-pentamethoxy-7,9′:7′,9-diepoxylignane (**33**) as aglycone, as well as glycosylated lignans, namely liriodendrin (**34**) and pinoresinol-4′-*O*-β-*D*-glucopyranoside (**35**) [54].

#### 2.1.6. Phytosterols

Stigmasterol (**36**) was isolated from the dichloromethane extract of *D. anethifolia* seeds [50], while 3-*O*-glucopyranosyl-β-sitosterol (**37**) was extracted from the ethyl acetate extract of the leaf and stem samples [49]. Ursodeoxycholic acid (**38**) as a sterol derivative (1.39%) was identified in the ethyl acetate extract of *D. anethifolia*’s aerial parts [49].

#### 2.1.7. Miscellaneous Compounds

Two phenolic glycosides (citrusin C (**39**) and coniferin (**40**)), beside the pterocarpan glycoside glycinol-3-*O*-β-d-glucopyranoside (**41**) as a novel phytoalexin, were isolated from the aqueous extract of *D. ismaelis’* aerial parts [54]. Vanillic aldehyde (**43**), 3-hydroxy-α-ionone (**45**) and 2-*C*-methyl-erythrytol (**47**) were isolated from *D. anethifolia*’s aerial parts (chloroform extract) [2].

Morgan et al., (2014) analyzed the aqueous fractions of *D. ismaelis’* aerial part extracts, and isolated blumenol-*C*-glucoside (**46**), the phytoestrogen coumestrol (**42**) and the hydroxycinnamic acid derivative (*Z*)-plicatin B (**44**) [54]. Succinic anhydride (**48**) and deoxyspergualin (**49**) were further specified as the predominant phytoconstituents of *D. anethifolia*’s aerial parts (3.37% and 2.08%, respectively) [49].

### 2.2. Volatile Components

The essential oil (EO) compositions of different plant parts (aerial part, fruit, flower, leaf and stem) of *D. anethifolia*, *D. assadii*, *D. flabellifolia* and *D. ismaelis*, collected from Iran, Saudi Arabia and Jordan, have been analyzed (Table 3).

Most of the studies were carried out on *D. anethifolia* harvested from Iran. Overall, the highest EO yield in the genus was observed in fresh aerial parts (2% (*v/w*) [19], while the lowest content was reported in the dried aerial parts of the same species (0.15% (*w*/*w*) [45]. Although the EOs have been mostly extracted by hydro-distillation (HD), steam distillation (SD), supercritical fluid (SFE) and solid phase micro extraction (SPME) were also applied. In a comparison study, the leaves of *D. anethifolia* were extracted by three methods, and it was observed that the highest yield could be obtained by SFE (1.7% (*w*/*w*), compared to HD and SD (0.53% and 0.89% (*w*/*w*), respectively) [46]. 

**Figure 2:**
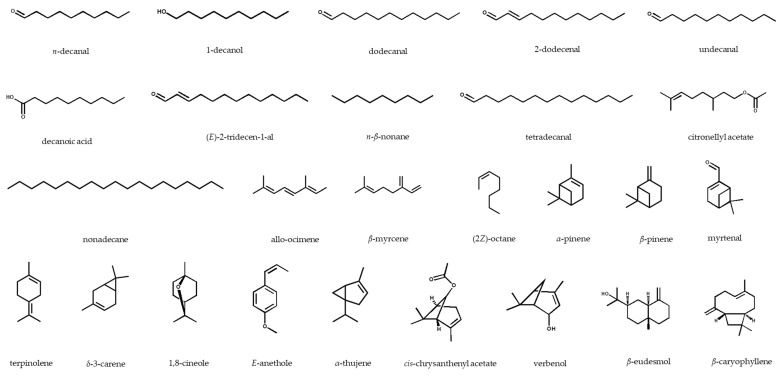
Chemical structures of the major essential oil compounds identified in the *Ducrosia* genus.

In general, aldehyde hydrocarbons, including decanal and dodecanal, were identified as the main aromatic compounds in the *Ducrosia* species. The highest content of decanal was reported in the aerial parts of *D. assadii* harvested from Lalehzar, Iran (74.0%) [14], whereas the EO from the aerial parts of the same plant collected from Dehbakri, Iran, contained only 10.1% [40]. Dodecanal was found in almost all the studied species, and it is the second major EO compound, with the highest amount detected in the fresh flowers of the EO of a Jordanian *D. flabellifolia* sample (33.41%) [13]. *cis*-Chrysanthenyl acetate (3.2‒72.28%) was characterized in *D. anethifolia* [43,44,45,47,58,59] and *D. assadii* [14,39,40]; furthermore, α-pinene as a monoterpene hydrocarbon was identified in all the species, with highest and lowest amounts in the leaves of *D. anethifolia* (70.3%) [15] and *D. assadii*’s aerial parts (4.0%), respectively [14]. The chemical structures of the main volatile compounds of the *Ducrosia* genus are displayed in Figure 2.

## 3. Pharmacological Activities

The crude extracts, EOs and isolated phytoconstituents of the *Ducrosia* species have been subjected to diverse in vitro and in vivo bioactivity assays. The most thoroughly studied species was *D. anethifolia* [2,8,15,18,19,20,48,49,50,51,58,59,60,61,62,63,64,65,66,67,68], however other species, such as *D. ismaelis* [42,52,60], *D. assadii* [26] and *D. flabellifolia* [8,61], have also been tested.

In one clinical trial performed on 90 patients, the anti-anxiety effect of a *D. anethifolia* EO-containing capsule with 50 mg of plant material was assessed in patients after acute myocardial infarction. Using the State-Trait Anxiety Inventory (STAI), the anti-anxiety effect was reported 96 h after taking the medication orally (twice a day), with a mean score of 33.35 ± 6.23 in the intervention group, while the anxiety rate was 36.48 ± 5.33 in the control group [62]. This is the only clinical trial carried out with *Ducrosia*-based products.

The antiradical, antimicrobial, antiproliferative and cytotoxic effects were the most extensively assessed bioactivities. In vivo studies mainly analyzed *D. anethifolia* EO for its analgesic, anti-anxiety, sedative, anti-inflammatory, immunostimulatory and neuroprotective activities, EO and extract for their anticonvulsant effect, extracts and isolated compounds for their antidiabetic potential, and extracts for their effects on testosterone hormone level. The anti-osteoporotic activity of isolated compounds from *D. ismaelis* has also been reported.

In the in vitro experiments, EO, extracts and isolated compounds of *D. anethifolia* exhibited antimicrobial, antioxidant, antiproliferative, cytotoxic and antidiabetic effects. The antioxidant activities of *D. ismaelis* (EO and isolated compounds), *D. assadii* (EO) and *D. flabellifolia* (EO and extract), besides the antimicrobial effects of EO and extracts from *D. ismaelis*, have also been reported (Table S1 in Supplementary Materials).

### 3.1. Analgesic Activity

The in vivo analgesic activity of EO extracted from *D. anethifolia* leaves has been evaluated in 84 male mice. Dose-dependent effects were observed using three assays, where the plant samples at 300 mg/kg had the highest potency, compared to 30 and 100 mg/kg, in the delayed response of mouse tail-flick (5.8/s), writhing (15 number) and pain scores in acute (0.8 score) and chronic phases (0.7 score), assessed by tail-flick, writhing and formalin methods, compared to morphine (1 mg/kg). The abovementioned parameters of 8.0/s, 2.5 number and 0.5 and 0.4 scores, respectively, were applied as positive control [63].

### 3.2. Anti-Anxiety and Sedative

The EO of *D. anethifolia*’s aerial parts was tested in the elevated plus maze test on mice. The percentage of time spent in the open arms within 5 min of administration to the animals was investigated.

The time spent in the open arm was 29% at 25 and 50 mg/kg doses of the EOs, while at 400 mg/kg, the percentage of entries into the open arms was 22%, and the efficacy was inferior to diazepam at 3 mg/kg, with 30% and 22%, respectively [18].

In this study, the response of spontaneous locomotor activity to the various EOs doses was also analyzed, during a 15 min period at 5 min of intervals. The treatments with 200 and 400 mg/kg of Eos both resulted in lower activity in the animals, compared to diazepam (3 mg/kg) [18].

### 3.3. Anticonvulsant Activity

The hydro-ethanolic extract (20%) from *D. anethifolia*’s aerial parts was assayed for its anticonvulsant effect on Wistar rats after inducing seizures by pentylenetetrazol (80 mg/kg). The plant extract at 2 mg/kg shortened the total length of seizure, which was 148.75 s, whilst its effect was inferior to diazepam (1 mg/kg), with a 19.37 s seizure [64].

In a similar study, the anticonvulsant effects of EO obtained from the aerial parts, and α-pinene as the major volatile component, of *D. anethifolia* were tested on Wistar rats suffering pentylenetetrazol (80 mg/kg)-induced seizures. The highest protection (100%) was recorded after the administration of 50 mg/kg EO, whereas diazepam showed complete protection (100%) at 2 mg/kg. The duration of the tonic seizure was the lowest with the application of 0.4 mg/kg of α-pinene, at 8 s, compared to EOs at doses of 25, 50, 100 and 200 mg/kg, while diazepam (2 mg/kg) was more active, with 4 s seizures [65].

### 3.4. Antidiabetic Activity

In a previous comprehensive study, the antidiabetic potency of *D. anethifolia* samples was evaluated in both in vitro and in vivo, and the results demonstrated promising activity. Among the leaf and stem extracts, and the isolated furanocoumarins, which were applied in concentrations of 10–100 µg/mL, imperatorin (**12**) and the crude extract showed the most potent inhibition of α-amylase and α-glucosidase enzymes. Imperatorin (**12**) inhibited the enzyme α-amylase with an effect (52.26% at 500 µg/mL) similar to that of the positive control, acarbose (52.55% at 500 µg/mL). The extract and imperatorin (**12**) inhibited α-glucosidase enzyme activity at a concentration of 10 µg/mL, with inhibition of 28.89%, compared to acarbose (at 10 µg/mL) with 29.94% [48].

In a further study, albino rats with streptozotocin-induced diabetes were treated for 45 days with 500 mg/kg hydro-ethanolic extract (80%), obtained from *D. anethifolia* leaf and stem. In comparison to the glibenclamide group at 5 mg/kg, with a blood glucose level of 151.50 mg/dL, the plant extract showed a good effect in decreasing the blood glucose level, with the level measured at 165.60 mg/dL [48].

### 3.5. Anti-Inflammatory Activity

Measured using a xylene-induced ear edema assay, the EO from *D. anethifolia*’s aerial parts (100 mg/kg) exhibited anti-inflammatory effects on mice with efficacy similar to dexamethasone (15 mg/kg), as determined by their reducing of the ear edema to values of 3.8% and 3.2%, respectively, compared to the control group [63].

### 3.6. Antimicrobial Activities

EOs, extracts and pure compounds of *D. anethifolia*, and the EOs and extracts of *D. ismaelis’* aerial parts have been investigated for antimicrobial activities.

Habibi et al., (2017) studied the EO of the aerial parts of *D. anethifolia* to evaluate its efficacy against some food-borne pathogens. The highest inhibitory effect was recorded against *Bacillus cereus*, by disk diffusion (DD) and microbroth dilution (MbD) assays, with an inhibition zone (IZ) of 13.33 mm, a minimum inhibitory concentration (MIC) of 7.81 mg/mL, and a minimum bactericidal concentration (MBC) of 31.25 mg/mL. These activities were comparable to tetracycline being used as the antibacterial drug, which possessed an IZ of 18 mm, an MIC of 7.81 mg/mL, and an MBC of 15.62 mg/mL [58].

In another study, the EO obtained from *D. anethifolia*’s aerial parts exhibited a moderate potency against the Gram-negative bacterium *Proteus vulgaris*, experimented via DD (IZ: 26.6 mm) and MbD (MIC: 0.39 mg/mL, MBC: 1.56 mg/mL), however it was less effective than tetracycline as the positive control (IZ: 32.6 mm, MIC: 0.19 mg/mL, MBC: 0.39 mg/mL) [59].

By applying a DD assay, the hydro-methanolic extract (90%) from the aerial parts of *D. anethifolia*, in different concentrations (0.5–4 mg/disk), was analyzed to assess its antimicrobial activities against two Gram-positive (*B. subtilis*, *Staphylococcus aureus*), two Gram-negative (*Escherichia coli*, *Pseudomonas aeruginosa*) bacteria, and one pathogenic yeast (*Candida albicans*). The extract at a concentration of 4 mg/disk possessed antimicrobial effects with an IZ of 10.7 mm against *E. coli*, while gentamicin (10 µg/disk) was more active, with a 15 mm inhibitory zone. Furthermore, against *C. albicans*, the extract in concentrations of 0.5‒2 mg/disk and 4 mg/disk showed activities with 6.4 and 6.7 mm, respectively, compared to ketoconazole (10 µg/disk), which had a 10 mm inhibition zone [66].

Two assays of MbD and DD were utilized to investigate the antibacterial potential of *D. anethifolia* EO and decanal, as its main component, against clinically isolated methicillin-resistant and methicillin-susceptible *S. aureus*. Overall, the EO was the stronger agent in inhibiting the bacteria, compared to the decanal. The EO had inhibition zones of 10.6 and 24.86 mm and minimum inhibition concentrations of 31.25 and 62.5 µg/mL, assessed by DD and MbD methods, respectively. The EO also had a good synergistic effect with methicillin [20].

Pangelin (syn. pabulenol) (**16**), a prenylated furanocoumarin isolated from *D. anethifolia*, exhibited weak anti-mycobacterial activity, with MIC values of 128 µg/mL against *Mycobacterium fortuitum* and 64 µg/mL against *M. smegmatis*, *M. phlei* and *M. aurum*. Ethambutol and isoniazid, on the other hand, were more potent as positive controls, with MICs of 0.25–8 µg/mL, and 0.5–2 µg/mL, respectively, evaluated by MbD assay [52].

The antimicrobial potency of EO extracted from the herb and fruits of *D. anethifolia*, along with its main oxygen-containing aliphatic constituents (*N*-decanal, *N*-dodecanal, *N*-decanol, *N*-dodecanol and trans-2-dodecenal), using a DD assay against several Gram-positive and Gram-negative bacteria, yeast and fungi strains, was tested. No inhibitory effect was detected against the two Gram-negative bacteria *P. aeruginosa* and *E. coli*; nevertheless, several samples demonstrated high activities. These included the EO of the herb against *Trichophyton rubrum* and *Epidermophyton floccosum* (IZ: 88 mm), and trans-2-dodecenal against *T. rubrum* (IZ: 86 mm) compared to griseofulvin (50 mg/mL, IZ: 26 mm, 32 mm, respectively); also *n*-decanal, *n*-decanol (IZ: 88 mm) and *n*-dodecanal (IZ: 94 mm) against *C. albicans*, compared to nystatin (50 mg/mL, IZ: 13 mm) [19].

In an in vivo study, the aqueous extract of *D. ismaelis* was orally administered to female albino mice. The mycotoxin-producing liquid culture of *Aspergillus flavus* (50 mL) was administered instead of water to a group of animals. Low fungal contamination, with total fungi counts of 250 colony-forming unit/g, was observed in the animals treated with the plant extract [60]. The EO yielded by the aerial parts of *D. ismaelis* was subjected to MbD assay to evaluate its antimicrobial effect. The remarkable effects of the EO were reported, particularly against the Gram-positive bacteria *S. aureus* and *S. epidermidis*, with MIC and MBC values of 0.07 and 0.15 mg/mL, compared to gentamycin (MIC and MBC values of 7.8 and 15.6 mg/mL, respectively). Moreover, the EO showed good inhibitory activity against *C. albicans* and *Rhodotorula* sp., with the minimum fungicidal concentration (MFC) 0.62 mg/mL, and against fungi *Aspergillus ochraceus* and *Penicillium chrysogenum* (MFC: 0.31 mg/mL), while the positive control nystatin was less potent, with an MFC value of 7.0 mg/mL [42].

### 3.7. Anti-Osteoporotic Activity

The in vitro anti-osteoporotic activities of some secondary metabolites of *D. ismaelis* were determined by utilizing the TRAP (tartrate-resistant acid phosphatase) method. *(Z)*-Plicatin B (**44**), citrusin C (**39**), daidzein-4′-*O*-β-d-glucopyranoside (**22**) and liriodendrin (**34**) suppressed osteoclast formation with TRAP values of 86.05%, 100.93%, 104.77% and 106.05% of the control, respectively, at a concentration of 10 µM, considering that daidzein as the positive control had an inhibition of 131% at the same concentration [54].

### 3.8. Antioxidant Activities

The free radicals scavenging potentials of the EOs from the flowers and fruits of *D. assadii* have been evaluated using the DPPH (2,2-diphenyl-1-picrylhydrazyl) assay. The activities were almost similar, the highest activities being recorded at 320 µL/mL EO from flowers and fruits, with 68% and 69% inhibitions, respectively [26].

So far, only methanolic and ethanolic extracts of *D. anethifolia* have been analyzed for antiradical activity. In a study, the hydro-methanolic extract (80%) of the aerial parts exhibited antiradical activity with an IC_50_ value of 0.38 g/L and an EC_50_ of 0.63 g/L, in the DPPH and FRAP (ferric reducing antioxidant of potency) assays, respectively, while ascorbic acid and quercetin as positive controls showed higher activities in both assays (IC_50_ of 0.033 and 0.017 g/L in DPPH, and EC_50_ of 0.091 and 0.026 g/L in FRAP, respectively) [49].

The antioxidant capacity of the hydro-methanolic extract (80%) of *D. anethifolia* was measured by applying several methods. Overall, the plant sample revealed a more potent antioxidant activity compared to the controls. It is noteworthy that, in the DPPH and FRAP assays, the IC_50_ values of the extract were calculated as 15.22 and 17.02 µg/mL, respectively, while the standards BHT (butylated hydroxytoluene) and ascorbic acid were less active, with IC_50_ values of 17.29 and 58.91 µg/mL, and 16.25 and 68.76 µg/mL, respectively [67]. In a similar work, DPPH results demonstrated the higher activity of the ethanolic extract of *D. anethifolia* leaf, with an IC_50_ of 122.02 ppm, compared to the ethyl acetate fraction (IC_50_: 354.37 ppm); however, both were less potent than BHT (IC_50_ 45.64 ppm) [15].

ORAC (oxygen radical absorbance capacity) and CUPRAC (copper reducing capacity) assays were used to analyze the radical-scavenging potentials of the isolated constituents of *D. ismaelis* at two concentrations (1 and 10 µM). The most active radical scavengers were genistin (**21**), at 10 µM with 27 µM Trolox equivalent (TE) in the ORAC assay, and 4′-hydroxy-3,3′,4,5,5′-pentamethoxy-7,9′:7′,9-diepoxylignane (**33**) at 24 µM TE in the CUPRAC assay [54].

The total antioxidant capacity of the EO from aerial parts of *D. ismaelis* was investigated by β-carotene bleaching assay. In comparison to rutin (positive control), whose capacity was 91.2%, the EO indicated a 68.5% capacity in the same concentration of 1 mg/mL. The highest inhibition of the EO was also seen for 1 mg/mL, at 72.1%, whilst ascorbic acid had a 94.2% inhibition, evaluated by DPPH method [42].

### 3.9. Antiproliferative and Cytotoxic Activities

Eight linear furanocoumarins isolated from *D. anethifolia* were analyzed to assess their antiproliferative and cytotoxic effects in vitro on PAR (L5178Y PAR mouse T-cell lymphoma), MDR (L5178Y human ABCB1-transfected subline) and NIH/3T3 (mouse embryonic fibroblast) cell lines. Among all the compounds, oxypeucedanin (**3**) and heraclenin (**9**) had the highest activities against the cancer cell lines. Oxypeucedanin (**3**) possessed the highest antiproliferative effect on the PAR and MDR cells, with ED_50_ (the drug dose producing 50% of a maximal effect) values of 25.98 and 28.89 μM, however doxorubicin was more potent as the positive control, with ED_50_ 0.054 and 0.46 μM, respectively. The most cytotoxic compound against PAR and MDR cells was also oxypeucedanin (**3**), with ED_50_ 40.33 and 66.68 μM, although doxorubicin possessed greater activity, with ED_50_ values of 0.37 and 7.15 μM, respectively. Assessment via checkerboard combination assay revealed that oxypeucedanin (**3**) (ratio of 1:50) and heraclenin (**9**) (ratio of 4:100) had a slight synergistic effect with doxorubicin, with ED_50_ values of 0.85 and 0.88 μM, respectively [2].

The in vitro cytotoxic potential of secondary metabolites isolated from the dichloromethane extract of *D. anethifolia* seeds was analyzed by applying an MTT (3-(4,5-dimethylthiazol-2-yl)-2,5-diphenyltetrazolium bromide) assay, which is a colorimetric method for evaluating cell metabolic activity. Ducrosin B (**28**) possessed the highest potential against human colon HCT-116 and ovarian carcinoma SKOV-3 cells, with IC_50_ 41.0 and 54.0 µM, respectively, however those activities were lower than the anticancer drug tamoxifen, used as a positive control, with IC_50_ values of 1.0 and 1.4 µM, respectively [50].

EOs from Iranian *D. anethifolia* and *D. flabellifolia* were analyzed to assess cytotoxic activity via MTT assay on three cell lines, namely K562 (human chronic myelogenous leukemia), LS180 (human colon adenocarcinoma) and MCF-7 (human breast adenocarcinoma). Generally, the EO from *D. anethifolia* was more potent compared to *D. flabellifolia*, while the highest cytotoxicity was measured for the EO of *D. anethifolia* on the K562 cell line (IC_50_: 85.5 µg/mL); however, it was weaker than cisplatin as the positive control (IC_50_: 6.9 µg/mL) [8].

Another study evaluated the antiproliferative activities of three different extracts (ethanolic, chloroform and aqueous) of *D. flabellifolia* on three cell lines, HEp-2 (larynx carcinoma), MCF-7 and Vero (African green monkey kidney), using an MTT assay. Among the extracts, the hydro-ethanolic (95%) was the most active on all the tested cells. This extract exhibited a higher inhibitory capacity on Vero cell proliferation, with an IC_50_ of 87.50 µg/mL, compared to vincristine sulphate (IC_50_ > 90 μg/mL) [61].

The EO obtained from aerial parts of *D. ismaelis* showed cytotoxicity against the MCF-7 cell line, characterized by an IC_50_ value of 66.24 μg/mL, although vinblastine was more potent (1.8 μg/mL). On the HEpG2 (liver cancer) and LoVo (colon cancer) cell lines, weaker activities were observed [42].

### 3.10. Neuroprotective Activities

In an animal experiment using a Morris water maze test, the impact of EO from *D. anethifolia*’s aerial parts on spatial learning and memory in rats was evaluated. The EO at a dose of 0.5 mL/kg remarkably increased the escape latency after the 2nd, 3rd and 4th treatments. Furthermore, the latency to find the hidden platform was significantly reduced on all the days of treatment (at 0.25 mL/kg), except the first day. Besides, compared to the control group, the time needed and the distance travelled to the target zone were both increased after administering 0.5 mL/kg EO. In conclusion, the EO of *D. anethifolia*, at doses of 0.25 and 0.5 mL/kg after four days of treatment, demonstrated a good capacity for improving spatial learning and memory in rats [68], thus more clinical trials are required to further prove its efficacy.

### 3.11. Other Pharmacological Activities

In an in vivo study, the effect of *D. anethifolia* EO on the blood immune parameters of rainbow trout (*Oncorhynchus mykiss*) was investigated. No impacts on serum bactericidal, serum lysozyme or respiratory burst activities were observed [69].

The enzyme NAD(P)H quinone oxidoreductase 1 (NQO1) inducer activity of the methanolic extract of *D. anethifolia* (80%), gained from the aerial parts, was assessed against murine hepatoma (Hepa1c1c7) cells. In comparison to sulforaphane as the positive control, the extract demonstrated a higher effect, with CD (concentration that doubles the specific activity of NQO1) values of 35.45 µg/mL vs. of 32 µg/mL, and a maximum induction of 4.5-fold vs. 2.5-fold [70].

The influence on testosterone concentration of a hydro-ethanolic extract (80%) from *D. anethifola*’s aerial parts was assessed on Wistar rats using a radioimmunoassay. The plant extract significantly decreased testosterone levels (0.24 ng/mL in animals treated with 140 mg/kg extract, compared to 2.4 ng/mL in the control group) [71].

The hypolipidemic activity of EOs obtained from the leaf and stem of *D. anethifolia* was investigated on streptozotocin-induced diabetic rats. The results of the group treated with EO were comparable to those of the control groups treated with glibenclamide. The total cholesterol of the EO group was 87.0 ug/dL, while in the control group treated with glibenclamide, it was 84.69 ug/dL. Moreover, the triglyceride levels of the EO-treated group and the glibenclamide control group were 39.40 and 38.18 ug/dL, respectively. In the EO group, HDL (high-density lipoprotein) and LDL (low-density lipoprotein) cholesterol were 18.23 mg/dL and 39.96 ug/dL, respectively, whereas in the glibenclamide group, similar results were recorded (18.82 mg/dL and 38.23 ug/dL, respectively) [48].

## 4. Conclusions and Prospective

The *Ducrosia* genus includes six species, mainly growing in Asia, particularly Iran, Saudi Arabia, Afghanistan, Pakistan and Iraq. *D. anethifolia,* as the most common species, has been traditionally consumed as a remedy for anxiety and insomnia, and as analgesic in Iranian folk medicine. It should be noted that the herbs of this species have been used as food additives in cuisine. Further species of the *Ducrosia* genus (e.g., *D. flabellifolia* and *D. assadii*) have also been known for their use as anti-inflammatory, antiseptic, carminative and soporific agents in traditional medicine.

Two species, *D. anethifolia* and *D. ismaelis*, have been analyzed to isolate their non-volatile secondary metabolites. A total of 17 linear furanocoumarins, 8 flavonoids, 5 terpenoids, 3 phytosterols and 2 alkaloids have been identified as the major compounds of these *Ducrosia* species. Furanocoumarins, as the main constituents of the genus, were present at the highest concentrations in the chloroform extracts of the aerial parts, however their occurrence in different plant parts has not been comprehensively studied. In general, among all the isolated compounds, four constituents, including one dihydrochalcone glycoside ismaeloside A (**25**), one pterocarpan glycoside glycinol-3-*O*-β-D-glucopyranoside (**41**), a monoterpene ducrosin A (**26**) and one sesquiterpene ducrosin B (**28**), have been characterized as the novel secondary metabolites.

The plants of this genus contain remarkable amounts of volatile compounds, predominantly analyzed in the fresh aerial parts of *D. anethifolia*, but different plant parts of *D. assadii*, *D. flabellifolia* and *D. ismaelis* have also been tested for their essential oil composition. Overall, decanal and dodecanal (aldehyde hydrocarbons), *cis*-chrysanthenyl acetate and α-pinene (monoterpene hydrocarbon) have been identified as the major fragrant compounds of the *Ducrosia* genus.

Except for one clinical trial, all the pharmacological investigations into the *Ducrosia* genus have been carried out in vivo (analgesic, antidiabetic, antianxiety, etc.) or in vitro (e.g., antimicrobial, antioxidant, antiproliferative), specifically on *D. anethifolia* as the most renowned and available plant species.

The efficacy and safety of the plants have not been investigated yet in detail. Further experiments are necessary to support the rationale of the folk medicinal applications, and the safety, of *Ducrosia* species.

## Figures and Tables

**Table 1:** The ethnobotanical application of *Ducrosia* species.

Plant Species	Plant Part	Plant Preparation	Ethnobotanical Uses	Locality	References
*D. anethifolia*	AP	nd	relieve for headache, backache, colic pains, catarrh	Mountainous and flat areas in Africa, Iran, Afghanistan, Lebanon, Iraq, Pakistan	[4,15,16,17,18,19,20,21]
			treatment of inflammation of the inner wall of the nose	Mountainous and flat areas in Africa, Iran, Afghanistan, Lebanon, Iraq, Pakistan	[7]
			relaxation the mind and body, treatment of anxiety, depression, insomnia	Iran (Shahrekord, Kashan), Arabian countries	[17,21,22,23]
			analgesic	Iran, Afghanistan, Pakistan, Syria, Lebanon, Saudi Arabia	[4,17,18,20,25]
			treatment of cold	Saudi Arabia	[24]
				Iran	[7,17,21]
			relieve heartburn	Iran	[21]
			use as animal feed	Iran, Iraq, Pakistan, Afghanistan, Syria, Lebanon, and some of the Arabian countries	[30]
			use as flavoring agent in food	Iran, Afghanistan, Pakistan, Syria, Lebanon, Saudi Arabia	[4,23,26,27,28,29,30]
			use as insect and reptile repellent	Saudi Arabia	[24]
				Iran	[7,17,21]
	AP, L, S	decoction	carminative, lactiferous agent	nd	[31,32,33,34]
	Se	infusion	relieve colic pain in children	Afghanistan, Iran, Iraq, Pakistan	[19]
	nd	nd	improve odor of foods and drinks	Iran, Afghanistan, Pakistan, and the rest of Middle East	[17]
*D. assadii*	AP	nd	analgesic, treatment of headache, backache, colic, colds, use as flavoring additive in foods	Iran	[29,35]
	nd	nd	flavoring additive in foods, antispasmodic, and carminative	Iran (Kerman)	[23,26]
*D. flabellifolia*	AP	smoking	treatment of toothache, general pain, sedative	Iran, Saudi Arabia, Jordan	[13]
	L	smoking	treatment of catarrh, headache, backache, jaundice, relieve dental, general pains, sedative	Iran, Iraq, Syria, Saudi Arabia, Jordan	[36]
*D. ismaelis*	AP	nd	treatment of skin infections, use as insect and reptile repellents	Africa and Iran, Iraq, Syria, Pakistan, Afghanistan, and Arabian countries	[2,4,23,24,37]

AP: aerial parts; L: leaf; nd: not determined; S: stem; Se: seed.

**Table 2:** The non-volatile phytoconstituents identified from *Ducrosia* species.

No.	Compound	Classification	*Ducrosia* Species	Plant Part	Plant Extract Fraction	Reference
1	Psoralen	L.F	*D. anethifolia*	L and S	EtOAc	[48]
2	5-Methoxypsoralen (syn. bergapten)	L.F	*D. anethifolia*	L and S	EtOAc	[48]
3	Oxypeucedanin	L.F	*D. anethifolia*	AP	CHCl_3_	[2]
4	Oxypeucedanin methanolate	L.F	*D. anethifolia*	AP	CHCl_3_	[2]
				L and S	EtOAc	[48]
5	8-Ethoxypsoralen	L.F	*D. anethifolia*	AP	EtOAc	[49]
6	Isogospherol	L.F	*D. anethifolia*	AP	CHCl_3_	[2]
7	Isooxypeucedanin	L.F	*D. anethifolia*	L and S	EtOAc	[48]
8	8-Methoxypsoralen (syn. xanthotoxin)	L.F	*D. anethifolia*	L and S	EtOAc	[48]
9	Heraclenin	L.F	*D. anethifolia*	AP	CHCl_3_	[2]
				Se	DCM	[50]
10	Prangenin (syn. oxyimperatorin)	L.F	*D. anethifolia*	AP	EtOAc	[49]
11	Heraclenol	L.F	*D. anethifolia*	AP	CHCl_3_	[2]
				Se	DCM	[50]
12	Imperatorin	L.F	*D. anethifolia*	AP	CHCl_3_	[2]
				L and S	EtOAc	[48]
13	Oxypeucedanin ethanolate	L.F	*D. ismaelis*	AP	CHCl_3_	[51]
14	(+)-Oxypeucedanin hydrate (syn. aviprin)	L.F	*D. anethifolia*	L and S	EtOAc	[48]
				AP	CHCl_3_	[2]
				Se	DCM	[50]
			*D. ismaelis*	AP	CHCl_3_	[51]
15	(‒)-Oxypeucedanin hydrate (syn. prangol)	L.F	*D. anethifolia*	AP	CHCl_3_	[2]
16	Pabulenol (syn. pangelin)	L.F	*D. anethifolia*	AP	CHCl_3_	[2,52]
				L and S	EtOAc	[48]
17	Saxalin	L.F	*D. ismaelis*	AP	CHCl_3_	[51]
18	Quercetin 3-glucoside	flavonol	*D. ismaelis*	L and S	EtOH (70%)	[53]
19	Quercetin 3-polyglycoside	flavonol	*D. ismaelis*	L and S	EtOH (70%)	[53]
20	Daidzin	isoflavone	*D. ismaelis*	AP	Aqueous	[54]
21	Genistin	isoflavone	*D. ismaelis*	AP	Aqueous	[54]
22	Daidzein-4′-*O*-β-d-glucopyranoside	isoflavone	*D. ismaelis*	AP	Aqueous	[54]
23	Prunetrin	isoflavone	*D. ismaelis*	AP	Aqueous	[54]
24	Isobavachalcone	chalcone	*D. ismaelis*	AP	Aqueous	[54]
25	Ismaeloside A	chalcone	*D. ismaelis*	AP	Aqueous	[54]
26	Ducrosin A	terpene	*D. anethifolia*	Se	DCM	[50]
27	8-*O*-Debenzoylpaeoniflorin	terpene	*D. anethifolia*	AP	CHCl_3_	[52]
28	Ducrosin B	terpene	*D. anethifolia*	Se	DCM	[50]
29	Isoaromadendrene epoxide	terpene	*D. anethifolia*	AP	EtOAc	[49]
30	Aromadendrene oxide	terpene	*D. anethifolia*	AP	EtOAc	[49]
31	Pseudosolasodine diacetate	alkaloid	*D. anethifolia*	AP	EtOAc	[49]
32	Harmine	alkaloid	*D. anethifolia*	AP	CHCl_3_	[2]
33	4′-Hydroxy-3,3′,4,5,5′- pentamethoxy-7,9′:7′,9-diepoxylignane	lignan	*D. ismaelis*	AP	Aqueous	[54]
34	Liriodendrin	lignan	*D. ismaelis*	AP	Aqueous	[54]
35	Pinoresinol-4′-*O*-β-d-glucopyranoside	lignan	*D. ismaelis*	AP	Aqueous	[54]
36	Stigmasterol	phytosterol	*D. anethifolia*	Se	DCM	[50]
37	3-*O*-glucopyranosyl-β-sitosterol	phytosterol	*D. anethifolia*	L and S	EtOAc	[48]
38	Ursodeoxycholic acid	phytosterol	*D. anethifolia*	AP	EtOAc	[49]
39	Citrusin C	Ph.G	*D. ismaelis*	AP	Aqueous	[54]
40	Coniferin	Ph.G	*D. ismaelis*	AP	Aqueous	[54]
41	Glycinol-3-*O*-β-d-glucopyranoside	P.G	*D. ismaelis*	AP	Aqueous	[54]
42	Coumestrol	phytoestrogen	*D. ismaelis*	AP	Aqueous	[54]
43	Vanillic aldehyde	P.A	*D. anethifolia*	AP	CHCl_3_	[2]
44	(*Z*)-Plicatin B	phenolic acid	*D. ismaelis*	AP	Aqueous	[54]
45	3-Hydroxy-α-ionone	A.M	*D. anethifolia*	AP	CHCl_3_	[2]
46	Blumenol-*C*-glucoside	F.A	*D. ismaelis*	AP	Aqueous	[54]
47	2-C-Methyl-erythrytol	A.A	*D. anethifolia*	AP	CHCl_3_	[2]
48	Succinic anhydride	C.D.A	*D. anethifolia*	AP	EtOAc	[49]
49	Deoxyspergualin	amide	*D. anethifolia*	AP	EtOAc	[49]

AP: aerial part; CHCl_3_: chloroform; DCM: dichloromethane; EtOAc: ethyl acetate; L: leaf; S: stem; Se: seed; Ph.G: phenolic glycoside; P.G: pterocarpan glycoside; P.A: phenolic aldehyde; L.F: linear furanocoumarin; A.M: apocarotenoid monoterpenoid; F.A: fatty acid; A.A: aliphatic alcohol; C.D.A: cyclic dicarboxylic anhydride.

**Table 3:** Volatile compositions characterized in *Ducrosia* species.

*Ducrosia* Species	Origin	Analyzed Plant Part	Stage/month of Sample Harvesting	Extraction Method	Yield (%)	Major Compounds and Chemical Classes (%)	References
*D. anethifolia*	Saudi Arabia (Riyadh province)	Fresh AP	nd	HD	nd	decanal (28.90), chrysanthenyl acetate (10.04), dodecanal (8.09) C.C: aldehyde hydrocarbons (39.21), oxygenated monoterpenes (14.49), monoterpenes hydrocarbons (11.60)	[43]
	Iran (Sistan and Balouchestan)	Dried AP	U.S/May 2015	HD	nd	chrysanthenyl acetate (41.54), decanal (19.55), methyl chavicol (9.25)	[44]
	Iran	AP	nd	HD	nd	*cis*-chrysanthenyl acetate (72.28), β-eudesmol (8.79), *E*-anethole (4.33)	[58]
	Iran (Bushehr)	Dried AP	nd	HD	nd	*cis*-chrysanthenyl acetate (72.28), 1,8-cineole (14.12), β-eudesmol (8.79)	[59]
	Iran (Kerman province)	Fresh AP	nd	HD	nd	*N-*decanal (70.1), α-pinene (12.40), β-caryophyllene (0.90)	[18]
	Iran (Karaj)	Fresh AP (L, S and Fr)	L and S: May-June I979 Fr: July I979	HD	2 (*v/w*)	α-pinene (59.2), *N-*decanal (52.2), *N-*dodecanal (20.5)	[19]
	Iran (Fars province)	Dried AP (S, L and Fl)	Fl.S/April 2015	HD	Kazeroun P: 0.43 ± 0.02 (*w*/*w*)	*N-*decanal (35.85), dodecanal (34.06), α-pinene (8.20)	[45]
					Noorabad P: 0.23 ± 0.09 (*w*/*w*)	*N-*decanal (29.44), dodecanal (21.83), (2e)-dodecenal (16.86)	
					Shiraz P-1: 0.15 ± 0.02 (*w*/*w*)	*N-*decanal (44.86), dodecanal (11.56), (2e)-dodecenal (5.74)	
					Shiraz P-2: 0.64 ± 0.02 (*w*/*w*)	*N-*decanal (18.81), dodecanal (16.64), decanoic acid (12.63)	
					Firoozabad P: 0.2 ± 0.03 (*w*/*w*)	*N-*decanal (34.74), dodecanal (11.69), limonene (1.49)	
					Farashband P: 0.45 ± 0.16 (*w*/*w*)	*N-*decanal (43.96), dodecanal (19.26), α-pinene (8.30)	
					Jahrom P-1: 0.55 ± 0.08 (*w*/*w*)	α-pinene (6.93), *cis*-chrysanthenyl acetate (6.73), *N-*decanol (5.63)	
					Jahrom P-2: 0.93 ± 0.04 (*w*/*w*)	*N-*decanol (49.22), α-pinene (16.54), undecanal (4.81)	
					Ghir P: 0.27 ± 0.14 (*w*/*w*)	*N-*decanal (41.49), dodecanal (18.58), α-pinene (6.27)	
					Darab P: 0.17 ± 0.03 (*w*/*w*)	*N-*decanal (24.58), α-pinene (14.17), dodecanal (11.69)	
	Iran (Tehran, Damavand)	L	Fl.S/June 2011	HD	0.53 (*w*/*w*)	*N-*decanal (30.31), dodecenal (14.35), *N-*decanol (11.04)	[46]
				SD	0.89 (*w*/*w*)	*N-*decanal (27.64), dodecanal (15.26), *N-*decanol (14.06)	
				SFE	1.7 (*w*/*w*)	*N-*decanal (25.67), dodecanal (17.25), dodecenal (16.23)	
	Iran (Kerman, Mehdi Abad)	Dried AP	Fl.S/June 2006	HD	nd	decanal (54.0), α-pinene (11.6), terpinolene (3.2)	[47]
	Iran (Fars, Neyriz)	Dried L	Fl.S/May 2012	HD	nd	α-pinene (70.3), β-myrcene (6.9), β-pinene (6.3)	[15]
	Iran (Fars, Larestan)	Dried AP	Fl.S	HD	0.35 (*w/w*)	decanal (18.8), α-thujene (14.5), decanol (9.3)	[38]
	Iran (West and Southwest)	Dried AP	Spring 2008	HD	nd	dodecanal (28.8), decanal (21.1), (E)-2-tridecen-1-al (15.8)	[8]
*D. assadii*	Iran (Kerman, Dehbakri)	Dried flowering and fruiting AP	Fl.S/June 2011	SD	nd	*N-*decanal (46.68), *N-*decanol (13.79), dodecanal (10.01)	[26]
			Fr.S/August 2011			*N-*decanal (42.21),*N-*decanol (13.27), dodecanal (8.87)	
						C.C:	
			Fl.S/June 2011	SD	nd	aliphatic (78.15), oxygenated aliphatic (76.88), monoterpenes (17.49)	
			Fr.S/August 2011			aliphatic (77.71), oxygenated aliphatic (77.25), monoterpenes (14.76)	
	Iran (Kerman, Lalehzar and Dehbakri)	Dried AP	Fl.S/July 2007	C.S-HD	nd	Lalehzar 1: decanal (74.0), dodecanal (7.2), α-pinene (4.0)	[14]
		Dried AP	Fl.S/July 2007	C.S-HD		Lalehzar 2: decanal (35.2), nonadecane (12), citronellyl acetate (11.6)	
		Dried AP	Fl.S/July 2007	C.S-HD		Dehbakrii: decanal (36.4), dodecanal (8.1), *cis*-chrysanthenyl acetate (6.7)	
	Iran (Kerman, Bakrii)	Dried AP	Fl.S/July 2002	HD	nd	decanal (36.4), dodecanal (8.1), *cis*-chrysanthenyl acetate (6.7)	[39]
	Iran (Kerman, Dehbakri)	Fresh AP	May and September 2007	HD	AP: 0.2 (*v/w*)	α-pinene (14.5), decanal (10.1), decanoic acid (10.4)	[40]
		fresh Fr		HD	Fr: 1.1 (*v/w*)	verbenol + myrtenal (71.0), allo-ocimene + δ-3-carene (4.0), crysanthenyl acetate (3.3)	
*D. flabellifolia*	Jordan (Northwest of the Nature Reserve Azraq)	Fresh L	Summer 2012	HD	nd	*N-*decanal (36.61), *N-*decanol (15.20), dodecanal (7.50)	[13]
		Dry L				*N-*decanal (21.00), dodecanal (8.29), *N-*β-nonane (7.06)	
		Fresh Fl				nd	
		Dry Fl				nd	
		Fresh L		SPME	nd	*N-*decanal (24.44), α-pinene (15.72), (2Z)-octane (7.04)	
		Dry L				*N-*decanol (27.88), β-pinene (13.56), terpinolene (10.51)	
		Fresh Fl				dodecanal (33.41), *N-*decanal (14.41), *n-*decanol (11.49)	
		Dry Fl				nd	
	Iran (west and southwest)	Dried AP	Spring 2008	HD	nd	decanal (32.8), dodecanal (32.6), decanol (4.3)	[8]
	Saudi Arabia (northern border)	S	nd	HD	nd	decanal (38.18), dodecanal (20.62), tetradecanal (6.31)	[41]
		L				decanal (50.02), dodecanal (14.82), α-pinene (4.36)	
		Fl				decanal (47.92), dodecanal (18.54), *N-*decanol (4.34)	
						C.C:	
		S				aldehyde hydrocarbons (65.11), oxygenated sesquiterpenes (10.83), monoterpenes hydrocarbons (2.48)	
		L				aldehyde hydrocarbons (65.39), monoterpenes hydrocarbons (9.37), oxygenated monoterpenes (3.35)	
		Fl				aldehyde hydrocarbons (67.30), monoterpenes hydrocarbons (5.53), oxygenated monoterpenes (5.15)	
*D. ismaelis*	Saudi Arabia (Riyadh)	Dried AP	January 2016	HD	nd	decanal (40.6), α-pinene (15.1), dodecanal (13.7) C.C: oxygenated monoterpenes (51.6)	[42]

## Supplementary Materials

The following are available online at https://www.mdpi.com/1424-8247/13/8/175/s1, Table S1: Pharmacological and biological activities of the *Ducrosia* genus.
