# Supplementary material for: Ducrosia spp., Rare Plants with Promising Phytochemical and Pharmacological Characteristics: An Updated Review
Source: Pharmaceuticals (Basel). 2020 Jul 31;13(8):175. doi: 10.3390/ph13080175 (PMC7464381; doi:10.3390/ph13080175)
Supplement: Supplementary file 1 [file pharmaceuticals-13-00175-s001.pdf]

Table S1. Pharmacological and biological activities of the *Ducrosia* genus

Activity	Plant	Assayed extract/plant product/compound	Measure of activity	Assay	Positive controls	Activity of controls	Cell lines/Strain/Model	References
Analgesic	*D. anethifolia*	EO of L (30 mg/kg)	DRTJ: 3 /s	Tail-flick	Morphine (1 mg/kg)	DRTJ: 8 /s	Mice (*in vivo*)	[60]
		EO of L (100 mg/kg)	DRTJ: 4.3 /s					
		EO of L (300 mg/kg)	DRTJ: 5.8 /s					
		EO of L (30 mg/kg)	Writhing: 29 no.	Writhing		Writhing: 2.5 no.		
		EO of L (100 mg/kg)	Writhing: 26 no.					
		EO of L (300 mg/kg)	Writhing: 15 no.					
		EO of L (30 mg/kg)	Pain Score:<br>AcP: 1.7 Sc<br>CP: 1.6 Sc	Formalin		Pain Score:<br>AcP: 0.5 sc.<br>CP: 0.4 sc.		
		EO of L (100 mg/kg)	Pain Score:<br>AcP: 1.8 sc.<br>CP: 0.9 sc.					
		EO of L (300 mg/kg)	Pain Score:<br>AcP: 0.8 sc.<br>CP: 0.7 sc.					

Antianxiety and sedative	*D. anethifolia*	EO of AP (2.5 mg/kg)	OAT: 20%	EPM	Diazepam (3 mg/kg)	OAT: 30%	Mice (*in vivo*)	[18]
		EO of AP (10 mg/kg)	OAT:20%					
		EO of AP (25 mg/kg)	OAT: 29%					
		EO of AP (50 mg/kg)	OAT: 29%					
		EO of AP (100 mg/kg)	OAT: 34%					
		EO of AP (200 mg/kg)	OAT: 40.5%					
		EO of AP (400 mg/kg)	OAT: 37%					
		EO of AP (2.5 mg/kg)	OAE: 10%	EPM	Diazepam (3 mg/kg)	OAE: 22%		
		EO of AP (10 mg/kg)	OAE:8%					
		EO of AP (25 mg/kg)	OAE: 19%					
		EO of AP (50 mg/kg)	OAE: 19%					
		EO of AP (100 mg/kg)	OAE: 37%					
		EO of AP (200 mg/kg)	OAE: 32%					
		EO of AP (400 mg/kg)	OAE: 22%					
		EO of AP (200 mg/kg)	LA: 14900 no. /15 min	LAA	Diazepam (3 mg/kg)	LA: 6000 no. /15 min		
		EO of AP (400 mg/kg)	LA: 13000 no. /15 min					
		EO of AP (200 mg/kg)	LS: 118 s	LAA	Diazepam (3 mg/kg)	LS: 90 s		
		EO of AP (400 mg/kg)	LS: 109 s					
		EO of AP (200 mg/kg)	ST: 1400 s		Diazepam (3 mg/kg)	ST: 1800 s		
		EO of AP (400 mg/kg)	ST: 1380 s					

Anticonvulsant	*D. anethifolia*	EtOH Ex. (20%) of AP (0.25 mg/kg)	LTBSA: 92.37 ± 21.50 s	RS	Diazepam (1 mg/Kg)	LTBSA: 172.37 ± 23.11 s	Wistar rats (*in vivo*)	[61]
		EtOH Ex. (20%) of AP (0.5 mg/kg)	LTBSA: 96.50 ± 16.00 s					
		EtOH Ex. (20%) of AP (1 mg/kg)	LTBSA: 101.4 ± 5.85 s					
		EtOH Ex. (20%) of AP (2 mg/kg)	LTBSA: 116.00 ± 18.80 s					
		EtOH Ex. (20%) of AP (0.25 mg/kg)	DLTS: 84.42 ± 9.26 s		Diazepam (1 mg/Kg)	DLTS: 13.25 ± 3.60 s		
		EtOH Ex. (20%) of AP (0.5 mg/kg)	DLTS: 67.75 ± 11.20 s					
		EtOH Ex. (20%) of AP (1 mg/kg)	DLTS: 61.93 ± 8.70 s					
		EtOH Ex. (20%) of AP (2 mg/kg)	DLTS: 54.37 ± 8.60 s					
		EtOH Ex. (20%) of AP (0.25 mg/kg)	DLTCS: 122.25 ± 16.80 s		Diazepam (1 mg/Kg)	DLTCS: 4.87 ± 2.20 s		
		EtOH Ex. (20%) of AP (0.5 mg/kg)	DLTCS: 126.25 ± 12.86 s					
		EtOH Ex. (20%) of AP (1 mg/kg)	DLTCS: 110.70 ± 13.60 s					
		EtOH Ex. (20%) of AP (2 mg/kg)	DLTCS: 96.17 ± 5.15 s					
		EtOH Ex. (20%) of AP (0.25 mg/kg)	TDSL: 305.60 ± 36.50 s		Diazepam (1 mg/Kg)	TDSL: 19.37 ± 4.20 s		
		EtOH Ex. (20%) of AP (0.5 mg/kg)	TDSL: 271.87 ± 22.70 s					
		EtOH Ex. (20%) of AP (1 mg/kg)	TDSL: 236.50 ± 22.60 s					
		EtOH Ex. (20%) of AP (2 mg/kg)	TDSL: 148.75 ± 23.90 s					
		EtOH Ex. (20%) of AP (0.25 mg/kg)	M: 49.4%		Diazepam (1 mg/Kg)	M: 0%		
		EtOH Ex. (20%) of AP (0.5 mg/kg)	M: 38.6%					
		EtOH Ex. (20%) of AP (1 mg/kg)	M: 22.5%					
		EtOH Ex. (20%) of AP (2 mg/kg)	M: 23.2%					

	EO of AP (25 mg/kg)	DMS: 20 ± 0.1 s	RS	Diazepam (2 mg/kg)	DMS: 2 ± 14 s	Wistar rats (*in vivo*)	[62]
	EO of AP (50 mg/kg)	DMS: 10 ± 42 s					
	EO of AP (100 mg/kg)	DMS: 13 ± 71 s					
	EO of AP (200 mg/kg)	DMS: 17 ± 42 s					
	α-pinene (0.2 mg/kg)	DMS: 16 ± 71 s					
	α-pinene (0.4 mg/kg)	DMS: 13 ± 43 s					
	EO of AP (25 mg/kg)	DTS: 40 ± 0.1 s		Diazepam (2 mg/kg)	DTS: 4 ± 85 s		
	EO of AP (50 mg/kg)	DTS: 13 ± 57 s					
	EO of AP (100 mg/kg)	DTS: 18 ± 14 s					
	EO of AP (200 mg/kg)	DTS: 19 ± 14 s					
	α-pinene (0.2 mg/kg)	DTS: 10 ± 57 s					
	α-pinene (0.4 mg/kg)	DTS: 8 ± 42 s					
	EO of AP (25 mg/kg)	DTCS: 162 ± 71 s		Diazepam (2 mg/kg)	DTCS: 4 ± 14 s		
	EO of AP (50 mg/kg)	DTCS: 13 ± 42 s					
	EO of AP (100 mg/kg)	DTCS: 35 ± 57 s					
	EO of AP (200 mg/kg)	DTCS: 27 ± 14 s					
	α-pinene (0.2 mg/kg)	DTCS: 31 ± 57 s					
	α-pinene (0.4 mg/kg)	DTCS: 21 ± 71 s					
	EO of AP (25 mg/kg)	M: 71%		Diazepam (2 mg/kg)	M: 0%		
	EO of AP (50 mg/kg)	M: 0%					
	EO of AP (100 mg/kg)	M: 14%					
	EO of AP (200 mg/kg)	M: 14%					
	α-pinene (0.2 mg/kg)	M: 42%					
	α-pinene (0.4 mg/kg)	M: 28%					

	EO of AP (25 mg/kg)	P: 29%	Diazepam (2 mg/kg)	P: 100%
	EO of AP (50 mg/kg)	P: 100%		
	EO of AP (100 mg/kg)	P: 86%		
	EO of AP (200 mg/kg)	P: 86%		
	α-pinene (0.2 mg/kg)	P: 58%		
	α-pinene (0.4 mg/kg)	P: 72%		

Antidiabetic	*D. anethifolia*	Ex. of L and St (10 μg/mL)	AI: 31.20 ± 2.34%	CMEI	Acarbose (10 μg/mL)	AI: 32.20 ± 1.29%	*in vitro*	[51]
		Psoralen (**1**) (10 μg/mL)	AI: 16.48 ± 0.98%					
		5-methoxypsoralen (**2**) (10 μg/mL)	AI: 17.59 ± 0.60%					
		Imperatorin (**12**) (10 μg/mL)	AI: 28.27 ± 2.97%					
		Isooxypeucedanin (**7**) (10 μg/mL)	AI: 19.21 ± 1.08%					
		Oxypeucedanin hydrate (**14**) (10 μg/mL)	AI: 18.40 ± 1.10%					
		Ex. of L and St (50 μg/mL)	AI: 29.33 ± 4.13%		Acarbose (50 μg/mL)	AI: 35.19 ± 34%		
		Psoralen (**1**) (50 μg/mL)	AI: 24.78 ± 2.24%					
		5-methoxypsoralen (**2**) (50 μg/mL)	AI: 28.70 ± 1.04%					
		Imperatorin (**12**) (50 μg/mL)	AI: 34.16 ± 1.04%					
		Isooxypeucedanin (**7**) (50 μg/mL)	AI: 22.11 ± 1.22%					
		Oxypeucedanin hydrate (**14**) (50 μg/mL)	AI: 23.33 ± 2.45%					
		Ex. of L and St (100 μg/mL)	AI: 45.00 ± 5.06%		Acarbose (100 μg/mL)	AI: 47.37 ± 4.15%		
		Psoralen (**1**) (100 μg/mL)	AI: 28.34 ± 1.89%					
		5-methoxypsoralen (**2**) (100 μg/mL)	AI: 34.29 ± 3.11%					
		Imperatorin (**12**) (100 μg/mL)	AI: 45.15 ± 6.18%					
		Isooxypeucedanin (**7**) (100 μg/mL)	AI: 24.14 ± 2.00%					
		Oxypeucedanin hydrate (**14**) (100 μg/mL)	AI: 25.30 ± 1.99%					
		Ex. of L and St (500 μg/mL)	AI: 49.97 ± 6.19%		Acarbose (500 μg/mL)	AI: 52.55 ± 4.49%		
		Psoralen (**1**) (500 μg/mL)	AI: 39.65 ± 2.80%					
		5-methoxypsoralen (**2**) (500 μg/mL)	AI: 45.12 ± 3.33%					
		Imperatorin (**12**) (500 μg/mL)	AI: 52.26 ± 5.69%					
		Isooxypeucedanin (**7**) (500 μg/mL)	AI: 39.77 ± 3.20%					
		Oxypeucedanin hydrate (**14**) (500 μg/mL)	AI: 37.08 ± 2.00%					

Ex. of L and St (1000 μg/mL)	AI: 70.77 ± 8.87%		Acarbose (1000 μg/mL)	AI: 71.34 ± 2.65%	
Psoralen (**1**) (1000 μg/mL)	AI: 50.50 ± 1.10%				
5-methoxypsoralen (**2**) (1000 μg/mL)	AI: 58.10 ± 2.14%				
Imperatorin (**12**) (1000 μg/mL)	AI: 67.56 ± 4.67%				
Isooxypeucedanin (**7**) (1000 μg/mL)	AI: 51.20 ± 3.40%				
Oxypeucedanin hydrate (**14**) (1000 μg/mL)	AI: 53.50 ± 1.10%				
Ex. of L and St (10 μg/mL)	GI: 28.89 ± 6.67%	GIA	Acarbose (10 μg/mL)	GI: 29.94 ± 2.04%	*in vitro*
Psoralen (**1**) (10 μg/mL)	GI: 23.00 ± 3.03%				
5-methoxypsoralen (**2**) (10 μg/mL)	GI: 25.15 ± 3.08%				
Imperatorin (**12**) (10 μg/mL)	GI: 28.89 ± 2.90%				
Isooxypeucedanin (**7**) (10 μg/mL)	GI: 24.33 ± 2.00%				
Oxypeucedanin hydrate (**14**) (10 μg/mL)	GI: 24.28 ± 2.13%				
Ex. of L and St (50 μg/mL)	GI: 40.20 ± 6.79%		Acarbose (50 μg/mL)	GI: 43.25 ± 3.09%	
Psoralen (**1**) (50 μg/mL)	GI: 33.55 ± 4.73%				
5-methoxypsoralen (**2**) (50 μg/mL)	GI: 34.81 ± 23.68%				
Imperatorin (**12**) (50 μg/mL)	GI: 37.76 ± 6.18%				
Isooxypeucedanin (**7**) (50 μg/mL)	GI: 32.12 ± 1.28%				
Oxypeucedanin hydrate (**14**) (50 μg/mL)	GI: 31.68 ± 5.00%				
Ex. of L and St (100 μg/mL)	GI: 47.30 ± 5.58%		Acarbose (100 μg/mL)	GI: 52.45 ± 4.67%	
Psoralen (**1**) (100 μg/mL)	GI: 41.65 ± 2.00%				
5-methoxypsoralen (**2**) (100 μg/mL)	GI: 45.20 ± 2.45%				
Imperatorin (**12**) (100 μg/mL)	GI: 46.56 ± 2.46%				
Isooxypeucedanin (**7**) (100 μg/mL)	GI: 39.90 ± 6.50%				

Oxypeucedanin hydrate (**14**) (100 μg/mL)	GI: 40.23 ± 4.56%			
Ex. of L and St (500 μg/mL)	GI: 56.25 ± 7.29%	Acarbose (500 μg/mL)	GI: 69.14 ± 4.15%	
Psoralen (**1**) (500 μg/mL)	GI: 42.75 ± 1.79%			
5-methoxypsoralen (**2**) (500 μg/mL)	GI: 48.00 ± 2.78%			
Imperatorin (**12**) (500 μg/mL)	GI: 55.67 ± 6.78%			
Isooxypeucedanin (**7**) (500 μg/mL)	GI: 41.51 ± 2.88%			
Oxypeucedanin hydrate (**14**) (500 μg/mL)	GI: 42.91 ± 2.60%			
Ex. of L and St (1000 μg/mL)	GI: 74.25 ± 8.09%	Acarbose (1000 μg/mL)	GI: 85.33 ± 2.47%	
Psoralen (**1**) (1000 μg/mL)	GI: 59.85 ± 2.90%			
5-methoxypsoralen (**2**) (1000 μg/mL)	GI: 64.39 ± 1.78%			
Imperatorin (**12**) (1000 μg/mL)	GI: 69.66 ± 3.67%			
Isooxypeucedanin (**7**) (1000 μg/mL)	GI: 52.31 ± 2.50%			
Oxypeucedanin hydrate (**14**) (1000 μg/mL)	GI: 51.67 ± 2.60%			
Ex. of L and St	BGL: 116.58 ± 4.00 mg/dL	Normal control	BGL: 111.53 ± 3.33 mg/dL	Albino rats (*in vivo*)
STZ + Ex. of L and St	BGL: 165.60 ± 8.30 mg/dL	STZ + Glibenclamide	BGL: 151.50 ± 2.10 mg/dL	
Ex. of L and St	AST: 2.43 ± 0.20 mg/mL	Normal control	AST: 2.55 ± 0.19 mg/mL	
STZ + Ex. of L and St	AST: 3.20 ± 0.21 mg/mL	STZ + Glibenclamide	AST: 2.85 ± 0.05 mg/mL	
Ex. of L and St	ALT: 1.52 ± 0.09 mg/mL	Normal control	ALT: 1.62 ± 0.028 mg/mL	
STZ + Ex. of L and St	ALT: 2.10 ± 0.23 mg/mL	STZ + Glibenclamide	ALT: 2.00 ± 0.16 mg/mL	
Ex. of L and St	HK: 102.00 ± 9.00 μmol/mg	Normal control	HK: 105.12 ± 1.67 μmol/mg	
STZ + Ex. of L and St	HK: 57.18 ± 0.95 μmol/mg	STZ + Glibenclamide	HK: 76.99 ± 8.89 μmol/mg	

		Ex. of L and St	PK: 64.69 ± 3.41 μmol/mg		Normal control	PK: 62.60 ± 2.45 μmol/mg		
		STZ + Ex. of L and St	PK: 47.47 ± 2.03 μmol/mg		STZ + Glibenclamide	PK: 50.16 ± 9.05 μmol/mg		
		Ex. of L and St	LDH: 39.00 ± 5.52 μmol/mg		Normal control	LDH: 39.27 ± 9.80 μmol/mg		
		STZ + Ex. of L and St	LDH: 29.90 ± 5.90 μmol/mg		STZ + Glibenclamide	LDH: 31.40 ± 8.48 μmol/mg		
		Ex. of L and St	PEPCK: 3.76 ± 0.45 μmol/mg		Normal control	PEPCK: 3.40 ± 0.30 μmol/mg		
		STZ + Ex. of L and St	PEPCK: 2.58 ± 0.23 μmol/mg		STZ + Glibenclamide	PEPCK: 2.87 ± 0.28 μmol/mg		
		Ex. of L and St	TU: 34.20 ± 3.80 mg/dL		Normal control	TU: 36.00 ± 6.10 mg/dL		
		STZ + Ex. of L and St	TU: 46.45 ± 3.07 mg/dL		STZ + Glibenclamide	TU: 33.23 ± 6.90 mg/dL		
		Ex. of L and St	Cr: 0.80 ± 0.05 mg/dL		Normal control	Cr: 0.84 ± 0.04 mg/dL		
		STZ + Ex. of L and St	Cr: 1.09 ± 0.12 mg/dL		STZ + Glibenclamide	Cr: 0.80 ± 0.04 mg/dL		
Anti-inflammatory	*D. anethifolia*	EO of AP (10 mg/kg)	ES: 7.1 ± 0.1%	nd	Dexamethasone (15 mg/kg)	ES: 3.2 ± 0.3%	Mice (*in vivo*)	[60]
		EO of AP (50 mg/kg)	ES: 4.1 ± 0.8%					
		EO of AP (100 mg/kg)	ES: 3.8 ± 0.1%					

Antimicrobial	*D. anethifolia*	EO of AP (0.98-250 mg/mL)	IZ: 13.33 ± 3.05 mm	DD	Tetracycline	IZ: 18 ± 4 mm	*B. cereus*	[58]
			IZ: 11.33 ± 1.15 mm			IZ: 35.33 ± 6.42 mm	*B. sphericus*	
			IZ: 17 ± 5 mm			IZ: 29 ± 2 mm	*B. anheracoid*	
			IZ: 0 mm			IZ: 26.66 ± 4.61 mm	*B. coagulance*	
			IZ: 0 mm			IZ: 18 ± 3.46 mm	*B. subtlis*	
			IZ: 19.66 ± 3.21			IZ: 30.66 ± 5.13 mm	*L. monocytogenes*	
			MIC: 7.8125 mg/mL	MbD		MIC: 7.8125 mg/mL	*B. cereus*	
			MIC: 15.625 mg/mL			MIC: 0.98 mg/mL	*B. sphericus*	
			MIC: 7.8125 mg/mL			MIC: 0.98 mg/mL	*B. anheracoid*	
			MIC: 125 mg/mL			MIC: 195 mg/mL	*B. coagulance*	
			no activity			MIC: 3.91 mg/mL	*B. subtlis*	
			MIC: 3.91 mg/mL			MIC: 0.98 mg/mL	*L. monocytogenes*	
			MBC: 31.25 mg/mL	MbD		MBC: 15.625 mg/mL	*B. cereus*	
			MBC: 31.25 mg/mL			MBC: 1.95 mg/mL	*B. sphericus*	
			MBC: 15.625 mg/mL			MBC: 3.91 mg/mL	*B. anheracoid*	
			no activity			MBC: 3.91 mg/mL	*B. coagulance*	
			no activity			MBC: 7.8125 mg/mL	*B. subtlis*	
			MBC: 15.625 mg/mL			MBC: 3.91 mg/mL	*L. monocytogenes*	

	EO of AP	IZ: 11 ± 1 mm	DD	Tetracycline	IZ: 33.3 ± 4.1 mm	*P. aeruginosa*	[59]
		IZ: 8.3 ± 1.5 mm			IZ: 32.3 ± 2.5 mm	*S. boydii*	
		IZ: 26.6 ± 3 mm			IZ: 32.6 ± 4.6 mm	*P. vulgaris*	
		MIC: 6.25 mg/mL	MbD		MIC: 0.195 mg/mL	*P. aeruginosa*	
		MIC: 25 mg/mL			MIC: 0.195 mg/mL	*S. boydii*	
		MIC: 0.39 mg/mL			MIC: 0.195 mg/mL	*P. vulgaris*	
		no activity	MbD		MBC: 0.39 mg/mL	*P. aeruginosa*	
		no activity			MBC: 0.78 mg/mL	*S. boydii*	
		MBC: 1.562 mg/mL			MBC: 0.39 mg/mL	*P. vulgaris*	

	MeOH Ex. (90%) of AP (0.5 mg/disk Ex.)	IZ: 6.4 mm	DD	Ampicillin (10 µg/disk)	IZ: 25 mm	*B. subtilis* (PTCC-1023)	[63]
		IZ: 6.4 mm			IZ: 35 mm	*S. aureus* (PTCC-1112)	
		IZ: 6.4 mm		Gentamicin (10 µg/disk)	IZ: 15 mm	*E. coli* (PTCC-1338)	
		IZ: 6.4 mm			IZ: 15 mm	*P. aeruginosa* (PTCC-	
		IZ: 6.4 mm		Ketoconazole (10 µg/disk)	IZ: 10 mm	1074)	
						C. albicans (PTCC-5027)	
	MeOH Ex. (90%) of AP (1 mg/disk Ex.)	IZ: 6.4 mm					
		IZ: 7.0 mm				*B. subtilis*	
		IZ: 7.5 mm				*S. aureus*	
		IZ: 6.7 mm				*E. coli*	
		IZ: 6.4 mm				*P. aeruginosa*	
						C. albicans	
	MeOH Ex. (90%) of AP (2 mg/disk Ex.)	IZ: 6.7 mm					
		IZ: 7.5 mm				*B. subtilis*	
		IZ: 8.5 mm				*S. aureus*	
		IZ: 7.7 mm				*E. coli*	
		IZ: 6.4 mm				*P. aeruginosa*	
						C. albicans	
	MeOH Ex. (90%) of AP (4 mg/disk Ex.)	IZ: 7.7 mm					
		IZ: 8.0 mm				*B. subtilis*	
		IZ: 10.7 mm				*S. aureus*	
		IZ: 8.07 mm				*E. coli*	
		IZ: 6.7 mm				*P. aeruginosa*	
						C. albicans	

	EO (2 μL)	IZ: 13.8 ± 0.58 mm	DD	Methicillin (5 μL)	IZ: MR	*S. aureus* (strain 3)	[20]
		IZ: 10.6 ± 1.15 mm			IZ: MR	*S. aureus* (strain 4)	
		IZ: 13.1 ± 0.28 mm			IZ: MR	*S. aureus* (strain 6)	
		IZ: 10.9 ± 1.04 mm			IZ: MR	*S. aureus* (strain 7)	
		IZ: 10.6 ± 0.28 mm			IZ: MS	*S. aureus* (strain 8)	
		IZ: 11.2 ± 0.28 mm			IZ: MR	*S. aureus* (strain 26)	
		IZ: 13.07 ± 1.00 mm			IZ: MS	*S. aureus* (strain 27)	
		IZ: 13.8 ± 0.76 mm			IZ: MR	*S. aureus* (strain 31)	
		IZ: 11.8 ± 2.6 mm			IZ: MR	*S. aureus* (strain 32)	
		IZ: 12.1 ± 0.76 mm			IZ: MS	*S. aureus* (strain 33)	
		IZ: 14.6 ± 0.58 mm			IZ: MR	*S. aureus* (strain 34)	
		IZ: 24.8 ± 0.76 mm			IZ: MS	*S. aureus* (strain 25923)	
		IZ: 10.8 ± 1.04 mm			IZ: MS	*S. aureus* (strain 6538)	
	EO (2 μL)	MIC: 62.5 μg/mL	MbD	nd	MIC: nd	*S. aureus* (strain 3)	
		MIC: 62.5 μg/mL				*S. aureus* (strain 4)	
		MIC: 62.5 μg/mL				*S. aureus* (strain 6)	
		MIC: 62.5 μg/mL				*S. aureus* (strain 7)	
		MIC: 62.5 μg/mL				*S. aureus* (strain 8)	
		MIC: 62.5 μg/mL				*S. aureus* (strain 26)	
		MIC: 62.5 μg/mL				*S. aureus* (strain 27)	
		MIC: 62.5 μg/mL				*S. aureus* (strain 31)	
		MIC: 62.5 μg/mL				*S. aureus* (strain 32)	
		MIC: 62.5 μg/mL				*S. aureus* (strain 33)	
		MIC: 62.5 μg/mL				*S. aureus* (strain 34)	
		MIC: 31.3 μg/mL				*S. aureus* (strain 25923)	
		MIC: 62.5 μg/mL				*S. aureus* (strain 6538)	

	EO (2 µL)	MBC: 125 µg/mL		nd	nd	*S. aureus* (strain 3)
		MBC: 125 µg/mL				*S. aureus* (strain 4)
		MBC: 125 µg/mL				*S. aureus* (strain 6)
		MBC: 62.5 µg/mL				*S. aureus* (strain 7)
		MBC: 125 µg/mL				*S. aureus* (strain 8)
		MBC: 125 µg/mL				*S. aureus* (strain 26)
		MBC: 62.5 µg/mL				*S. aureus* (strain 27)
		MBC: 125 µg/mL				*S. aureus* (strain 31)
		MBC: 125 µg/mL				*S. aureus* (strain 32)
		MBC: 62.5 µg/mL				*S. aureus* (strain 33)
		MBC: 125 µg/mL				*S. aureus* (strain 34)
		MBC: 62.5 µg/mL				*S. aureus* (strain 25923)
		MBC: 125 µg/mL				*S. aureus* (strain 6538)
	Decanal (2 µL)	IZ: No activity	DD	Methicillin (5 µL)	IZ: MR	*S. aureus* (strain 3)
		IZ: 7 ± 0.0 mm			IZ: MR	*S. aureus* (strain 4)
		IZ: 7 ± 0.0 mm			IZ: MR	*S. aureus* (strain 6)
		IZ: 8 ± 1.0 mm			IZ: MR	*S. aureus* (strain 7)
		IZ: 7 ± 0.0 mm			IZ: MS	*S. aureus* (strain 8)
		IZ: 7 ± 0.0 mm			IZ: MR	*S. aureus* (strain 26)
		IZ: No activity			IZ: MS	*S. aureus* (strain 27)
		IZ: 8 ± 0.0 mm			IZ: MR	*S. aureus* (strain 31)
		IZ: No activity			IZ: MR	*S. aureus* (strain 32)
		IZ: 8 ± 0.0 mm			IZ: MS	*S. aureus* (strain 33)
		IZ: 7 ± 0.0 mm			IZ: MR	*S. aureus* (strain 34)
		IZ: 8 ± 0.0 mm			IZ: MS	*S. aureus* (strain 25923)

		IZ: 8 ± 0.0 mm			IZ: MS	*S. aureus* (strain 6538)
	Decanal (2 μL)	MIC: 62.5 μg/mL	MbD	nd	nd	*S. aureus* (strain 3)
		MIC: 125 μg/mL				*S. aureus* (strain 4)
		MIC: 125 μg/mL				*S. aureus* (strain 6)
		MIC: 125 μg/mL				*S. aureus* (strain 7)
		MIC: 125 μg/mL				*S. aureus* (strain 8)
		MIC: 125 μg/mL				*S. aureus* (strain 26)
		MIC: 125 μg/mL				*S. aureus* (strain 27)
		MIC: 125 μg/mL				*S. aureus* (strain 31)
		MIC: 125 μg/mL				*S. aureus* (strain 32)
		MIC: 125 μg/mL				*S. aureus* (strain 33)
		MIC: 125 μg/mL				*S. aureus* (strain 34)
		MIC: 125 μg/mL				*S. aureus* (strain 25923)
		MIC: 125 μg/mL				*S. aureus* (strain 6538)
	Decanal (2 μL)	MBC: 125 μg/mL			nd	*S. aureus* (strain 3)
		MBC: 250 μg/mL				*S. aureus* (strain 4)
		MBC: 250 μg/mL				*S. aureus* (strain 6)
		MBC: 250 μg/mL				*S. aureus* (strain 7)
		MBC: 250 μg/mL				*S. aureus* (strain 8)
		MBC: 250 μg/mL				*S. aureus* (strain 26)
		MBC: 250 μg/mL				*S. aureus* (strain 27)
		MBC: 250 μg/mL				*S. aureus* (strain 31)
		MBC: 250 μg/mL				*S. aureus* (strain 32)
		MBC: 250 μg/mL				*S. aureus* (strain 33)
		MBC: 250 μg/mL				*S. aureus* (strain 34)

		MBC: 250 μg/mL				*S. aureus* (strain 25923)
		MBC: 250 μg/mL				*S. aureus* (strain 6538)
	EO (2 μL) + Methicillin (5 μL)	IZ: 16.6 ± 0.58 mm	DD	Methicillin (5 μL)	IZ: MR	*S. aureus* (strain 3)
		IZ: 12.6 ± 1.2 mm			IZ: MR	*S. aureus* (strain 4)
		IZ: 15.0 ± 0.0 mm			IZ: MR	*S. aureus* (strain 6)
		IZ: 24.6 ± 0.58 mm			IZ: MR	*S. aureus* (strain 7)
		IZ: 20.0 ± 1.0 mm			IZ: MS	*S. aureus* (strain 8)
		IZ: 25.0 ± 1.0 mm			IZ: MR	*S. aureus* (strain 26)
		IZ: 21.0 ± 1.4 mm			IZ: MS	*S. aureus* (strain 27)
		IZ: 17.3 ± 0.58 mm			IZ: MR	*S. aureus* (strain 31)
		IZ: 14.6 ± 1.15 mm			IZ: MR	*S. aureus* (strain 32)
		IZ: 15.3 ± 0.58 mm			IZ: MS	*S. aureus* (strain 33)
		IZ: 20.0 ± 0.58 mm			IZ: MR	*S. aureus* (strain 34)
		IZ: 28.0 ± 0.8 mm			IZ: MS	*S. aureus* (strain 25923)
		IZ: 29.5 ± 0.58 mm			IZ: MS	*S. aureus* (strain 6538)

	Pangelin (syn. pabulenol) (**16**)	MIC: 128 μg/mL	MbD	Ethambutol	MIC: 8 μg/mL	*Mycobacterium fortuitum* (ATCC-6841)	[54]
		MIC: 64 μg/mL			MIC: 0.25 μg/mL	*M. smegmatis* (ATCC-14468)	
		MIC: 64 μg/ mL			MIC: 1 μg/mL	*M. phlei* (ATCC-11758)	
		MIC: 64 μg/mL			MIC: 1 μg/mL	*M. aurum* (104482)	
				Isoniazid	MIC: 0.5 μg/mL	*M. fortuitum* (ATCC-6841)	
					MIC: 2 μg/mL	*M. smegmatis* (ATCC-14468)	
					MIC: 2 μg/mL	*M. phlei* (ATCC-11758)	
					MIC: 2 μg/mL	*M. aurum* (10448)	

	EO of herb	IZ: 70 ± 9 mm	DD	Streptomycin (10 mg/mL)	IZ: 21 ± 2 mm	*B. subtilis* (ATCC-6633)	[19]
		IZ: 29 ± 4mm		Streptomycin (10 mg/mL)	IZ: 17 ± 2 mm	*S. aureus* (ATCC-6538)	
		IZ: 0 mm		Streptomycin (10 mg/mL)	IZ: 16 ± 2 mm	*P. aeruginosa* (ATCC-	
		IZ: 0 mm		Streptomycin (10 mg/mL)	IZ: 16 ± 2 mm	9027)	
		IZ: 63 ± 16 mm		Nystatin (50 mg/mL)	IZ: 13 ± 1 mm	*E. coli* (ATCC-8739)	
		IZ: 84 ± 1 mm		Griseofulvin (50 mg/mL)	IZ: 20 ± 2mm	*C. albicans* (ATCC-10235)	
		IZ: 88 ± 0 mm		Griseofulvin (50 mg/mL)	IZ: 26 ± 5 mm	*T. mentagrophytes* var.	
		IZ: 88 ± 0 mm		Griseofulvin (50 mg/mL)	IZ: 32 ± 2 mm	*interdigitale* (CBS-558-66)	
						T. rubrum (CBS-392-58)	
	EO of fruit	IZ: 7 ± 1 mm				*E. floccosum* (CBS-108-67)	
		IZ: 7 ± 1 mm					
		IZ: 0 mm				*B. subtilis* (ATCC-6633)	
		IZ: 0 mm				*S. aureus* (ATCC-6538)	
		IZ: 7 ± 1 mm				*P. aeruginosa* (ATCC-	
		IZ: 26 ± 8 mm				9027)	
		IZ: 24 ± 7 mm				*E. coli* (ATCC-8739)	
		IZ: 23 ± 9 mm				*C. albicans* (ATCC-10235)	
						T. mentagrophytes var.	
	n-Decanal	IZ: 25 ± 12 mm				*interdigitale* (CBS-558-66)	
		IZ: 16 ± 3 mm				*T. rubrum* (CBS-392-58)	
		IZ: 0 mm				*E. floccosum* (CBS-108-67)	
		IZ: 0 mm					
		IZ: 88 ± 0 mm				*B. subtilis* (ATCC-6633)	
		IZ: 57 ± 13 mm				*S. aureus* (ATCC-6538)	
		IZ: 87 ± 3 mm				*P. aeruginosa* (ATCC-	
		IZ: 86 ± 2 mm				9027)	
						E. coli (ATCC-8739)	

	n-Dodecanal	IZ: 17 ± 2 mm	*C. albicans* (ATCC-10235)
		IZ: I8 ± 2 mm	*T. mentagrophytes* var.
		IZ: 0 mm	*interdigitale* (CBS-558-66)
		IZ: 0 mm	*T. rubrum* (CBS-392-58)
		IZ: 94 ± 2 mm	*E. floccosum* (CBS-108-67)
		IZ: 56 ± 15 mm	
		IZ: 60 ± 30 mm	*B. subtilis* (ATCC-6633)
		IZ: 78 ± 10 mm	*S. aureus* (ATCC-6538)
			P. aeruginosa (ATCC-
	n-Decanol	IZ: 15 ± 6 mm	9027)
		IZ: 17 ± 4mm	*E. coli* (ATCC-8739)
		IZ: 0 mm	*C. albicans* (ATCC-10235)
		IZ: 0 mm	*T. mentagrophytes* var.
		IZ: 88 ± 0 mm	*interdigitale* (CBS-558-66)
		IZ: 87 ± 2 mm	*T. rubrum* (CBS-392-58)
		IZ: 65 ± 14 mm	*E. floccosum* (CBS-108-67)
		IZ: 65 ± 15 mm	
			B. subtilis (ATCC-6633)
	n-Dodecanol	IZ: 8 ± 1 mm	*S. aureus* (ATCC-6538)
		IZ: 14 ± 6 mm	*P. aeruginosa* (ATCC-
		IZ: 0 mm	9027)
		IZ: 0 mm	*E. coli* (ATCC-8739)
		IZ: 10 ± 5 mm	*C. albicans* (ATCC-10235)
		IZ: 13 ± 4 mm	*T. mentagrophytes* var.
		IZ: 12 ± 2 mm	*interdigitale* (CBS-558-66)
		IZ: 9 ± 1 mm	*T. rubrum* (CBS-392-58)
			E. floccosum (CBS-108-67)

	trans-2-Dodecenal	IZ: 60 ± 1 mm<br>IZ: 25 ± 4 mm<br>IZ: 0 mm<br>IZ: 7 ± 1 mm<br>IZ: 13 ± 1 mm<br>IZ: 71 ± 11 mm<br>IZ: 86 ± 3 mm<br>IZ: 78 ± 10 mm				*B. subtilis* (ATCC-6633)<br>*S. aureus* (ATCC-6538)<br>*P. aeruginosa* (ATCC-9027)<br>*E. coli* (ATCC-8739)<br>*C. albicans* (ATCC-10235)<br>*T. mentagrophytes* var. *interdigitale* (CBS-558-66)<br>*T. rubrum* (CBS-392-58)<br>*E. floccosum* (CBS-108-67)<br><br>*B. subtilis* (ATCC-6633)<br>*S. aureus* (ATCC-6538)<br>*P. aeruginosa* (ATCC-9027)<br>*E. coli* (ATCC-8739)<br>*C. albicans* (ATCC-10235)<br>*T. mentagrophytes* var. *interdigitale* (CBS-558-66)<br>*T. rubrum* (CBS-392-58)<br>*E. floccosum* (CBS-108-67)	
D. ismaelis	Aqueous Ex. of AP	TFC: 250 CFU/g	CFUC	nd	nd	*Aspergillus flavus*/albino mice	[69]

	EO of AP	MIC: 0.07 mg/mL	MbD	Gentamycin (125 - 0.97 µg/mL)	MIC: 7.8 mg/mL	*S. aureus* (TCC-25923)	[42]
		MIC: 0.07 mg/mL		Gentamycin (125 - 0.97 µg/mL)	MIC: 7.8 mg/mL	*S. epidermidis* (ATCC-	
		MIC: 2.5 mg/mL		Gentamycin (125 - 0.97 µg/mL)	MIC: 3.9 mg/mL	1228)	
		MIC: 2.5 mg/mL		Gentamycin (125 - 0.97 µg/mL)	MIC: 3.9 mg/mL	*E. coli* (ATCC-25922)	
		MIC: 0.31 mg/mL		Nystatin (125 - 0.97 µg/mL)	MIC: 3.5 mg/mL	*Acintobacter* sp. (ATCC-	
		MIC: 0.15 mg/mL		Nystatin (125 - 0.97 µg/mL)	MIC: 3.5 mg/mL	49139)	
		MIC: 0.15 mg/mL		Nystatin (125 - 0.97 µg/mL)	MIC: 3.5 mg/mL	*C. albicans* (ATCC-60193)	
		MIC: 0.15 mg/mL		Nystatin (125 - 0.97 µg/mL)	MIC: 3.5 mg/mL	*Rhodotorula* sp.	
						A. ochraceus	
		MBC: 0.15 mg/mL		Gentamycin (125 - 0.97 µg/mL)	MBC: 15.6 mg/mL	*P. chrysogenum*	
		MBC: 0.15 mg/mL		Gentamycin (125 - 0.97 µg/mL)	MBC: 15.6 mg/mL		
		MBC: 5.0 mg/mL		Gentamycin (125 - 0.97 µg/mL)	MBC: 7.8 mg/mL	*S. aureus* (TCC-25923)	
		MBC: 5.0 mg/mL		Gentamycin (125 - 0.97 µg/mL)	MBC: 7.8 mg/mL	*S. epidermidis* (ATCC-	
						1228)	
		MFC: 0.62 mg/mL		Nystatin (125 - 0.97 µg/mL)	MFC: 7.0 mg/mL	*E. coli* (ATCC-25922)	
		MFC: 0.62 mg/mL		Nystatin (125 - 0.97 µg/mL)	MFC: 7.0 mg/mL	*Acintobacter* sp. (ATCC-	
		MFC: 0.31 mg/mL		Nystatin (125 - 0.97 µg/mL)	MFC: 7.0 mg/mL	49139)	
		MFC: 0.31 mg/mL		Nystatin (125 - 0.97 µg/mL)	MFC: 7.0 mg/mL		
						C. albicans (ATCC-60193)	
						Rhodotorula sp.	
						A. ochraceus	
						P. chrysogenum	

Anti-osteoporotic	*D. ismaelis*	CC: 1 μM		TRAP	Osteoblast induced by RANKL	I: 170%		[57]
		Glycinol-3-*O*-β-*D*-glucopyranoside (**41**)	I: 169%		(10 μM)		-	
		Ismaeloside A (**25**)	I: 180%		Daidzein (10 μM)	I: 131%		
		Daidzin (**20**)	I: 119%					
		Daidzein-4′-*O*-β-*D*-glucopyranoside (**22**)	I: 118%					
		Genistin (**21**)	I: 159%					
		Prunetrin (**23**)	I: 179%					
		Coumestrol (**42**)	I: 180%					
		Isobavachalcone (**24**)	I: 135%					
		4′-hydroxy-3,3′,4,5,5′-pentamethoxy-7,9′:7′,9-diepoxylignane (**33**)	I: 178%					
		Liriodendrin (**34**)	I: 128%					
		Pinoresinol-4′-O-β-D-glucopyranoside (**35**)	I: 180%					
		Blumenol-*C*-glucoside (**46**)	I: 132%					
		Citrusin C (**39**)	I: 120%					
		Coniferin (**40**)	I: 160%					
		(*Z*)-plicatin B (**44**)	I: 150%					
		CC: 10 μM						
		Glycinol-3-*O*-β-*D*-glucopyranoside (**41**)	I: 160%					
		Ismaeloside A (**25**)	I: 181%					
		Daidzin (**20**)	I: 119%					
		Daidzein-4′-*O*-β-*D*-glucopyranoside (**22**)	I: 102%					
		Genistin (**21**)	I: 181%					
		Prunetrin (**23**)	I: 150%					
		Coumestrol (**42**)	I: 170%					
		Isobavachalcone (**24**)	I: 117%					

	4′-hydroxy-3,3′,4,5,5′- pentamethoxy-7,9′:7′,9-diepoxylignane (**33**)	I: 178%
	Liriodendrin (**34**)	I: 112%
	Pinoresinol-4′-O-β-D-glucopyranoside (**35**)	I: 176%
	Blumenol-*C*-glucoside (**46**)	I: 170%
	Citrusin C (**39**)	I: 120%
	Coniferin (**40**)	I: 157%
	(*Z*)-plicatin B (**44**)	I: 86.05%

Antioxidant	*D. assadii*	EO of Fl (10 µL/mL)	I: 13%	DPPH	nd	nd	[26]
		EO of Fl (20 µL/mL)	I: 20%				
		EO of Fl (40 µL/mL)	I: 33%				
		EO of Fl (80 µL/mL)	I: 52%				
		EO of Fl (160 µL/mL)	I: 58%				
		EO of Fl (320 µL/mL)	I: 68%				
		EO of Fr (10 µL/mL)	I: 15%		nd	nd	
		EO of Fr (20 µL/mL)	I: 26%				
		EO of Fr (40 µL/mL)	I: 37%				
		EO of Fr (80 µL/mL)	I: 59%				
		EO of Fr (160 µL/mL)	I: 65%				
		EO of Fr (320 µL/mL)	I: 69%				
		EO of Fl (0 min)	DR: 100%		BHA	DR: 100% (0 min)	
		EO of Fl (5 min)	DR: 90%			DR: 19% (5 min)	
		EO of Fl (10 min	DR: 80%			DR: 19% (10 min)	
		EO of Fl (15 min)	DR: 70%			DR: 19% (15 min)	
		EO of Fl (20 min)	DR: 60%			DR: 19% (20 min)	
		EO of Fl (25 min)	DR: 45%			DR: 19% (25 min)	
		EO of Fl (30 min)	DR: 40%			DR: 19% (30 min)	
		EO of Fr (0 min)	DR: 100%		BHA	DR: 100% (0 min)	
		EO of Fr (5 min)	DR: 80%			DR: 19% (5 min)	
		EO of Fr (10 min	DR: 75%			DR: 19% (10 min)	
		EO of Fr (15 min)	DR: 70%			DR: 19% (15 min)	
		EO of Fr (20 min)	DR: 55%			DR: 19% (20 min)	

	EO of Fr (25 min)	DR: 40%			DR: 19% (25 min)	
	EO of Fr (30 min)	DR: 38%			DR: 19% (30 min)	
D. anethifolia	MeOH Ex. (80%) of AP	IC_{50}: 0.38 ± 0.02 g/L	DPPH	Ascorbic acid	IC_{50}: 0.033±0.001 g/L	[52]
		EC_{50}: 0.63 ± 0.03 g/L	FRAP	Ascorbic acid	EC_{50}: 0.091±0.002 g/L	
			DPPH	Quercetin	IC_{50}: 0.017 ± 0.001 g/L	
			FRAP	Quercetin	EC_{50}: 0.026 ± 0.002 g/L	
	MeOH Ex. (80%)	IC_{50}: 15.22 µg/mL	DPPH	BHT	IC_{50}: 17.29 µg/mL	[64]
				Ascorbic acid	IC_{50}: 16.25 µg/mL	
		IC_{50}: 17.02 µg/mL	FRAP	BHT	IC_{50}: 58.91 µg/mL	
				Ascorbic acid	IC_{50}: 68.76 µg/mL	
		IC_{50}: 17.05 µg/mL	SARS	BHT	IC_{50}: 43.83 µg/mL	
		IC_{50}: 6.91 µg/mL	NORS		IC_{50}: 17.75 µg/mL	
		IC_{50}: 7.07 µg/mL	HPS		IC_{50}: 15.59 µg/mL	
		IC_{50}: 18.34 µg/mL	TAC		IC_{50}: 28.35 µg/mL	
			SARS	Ascorbic acid	IC_{50}: 51.80 µg/mL	
			NORS		IC_{50}: 17.94 µg/mL	
			HPS		IC_{50}: 15.17 µg/mL	
			TAC		IC_{50}: 30.48 µg/mL	
	EtOH Ex. of L	IC_{50}: 122.02 ppm	DPPH	BHT	IC_{50}: 45.64 ppm	[15]
	EtOAc Ex. of L	IC_{50}: 354.37 ppm				

D. ismaelis	Glycinol-3-*O*-β-*D*-glucopyranoside (**41**)	4 μM TE	ORAC (1 μM)	nd	nd	[57]
	Ismaeloside A (**25**)	2 μM TE				
	Daidzin (**20**)	5 μM TE				
	Daidzein-4′-*O*-β-*D*-glucopyranoside (**22**)	4 μM TE				
	Genistin (**21**)	5.5 μM TE				
	Prunetrin (**23**)	2 μM TE				
	Coumestrol (**42**)	2 μM TE				
	Isobavachalcone (**24**)	5 μM TE				
	4′-hydroxy-3,3′,4,5,5′-pentamethoxy-7,9′:7′,9-diepoxylignane (**33**)	2.5 μM TE				
	Liriodendrin (**34**)	0 μM TE				
	Pinoresinol-4′-O-β-D-glucopyranoside (**35**)	4 μM TE				
	Blumenol-*C*-glucoside (**46**)	0.5 μM TE				
	Citrusin C (**39**)	0 μM TE				
	Coniferin (**40**)	0.1 μM TE				
	(*Z*)-plicatin B (**44**)	2.5 μM TE				
	Glycinol-3-*O*-β-*D*-glucopyranoside (**41**)	23 μM TE	ORAC (10 μM)	nd	nd	
	Ismaeloside A (**25**)	15.5 μM TE				
	Daidzin (**20**)	25.5 μM TE				
	Daidzein-4′-*O*-β-*D*-glucopyranoside (**22**)	20.5 μM TE				
	Genistin (**21**)	27 μM TE				
	Prunetrin (**23**)	12 μM TE				
	Coumestrol (**42**)	8.5 μM TE				
	Isobavachalcone (**24**)	25 μM TE				
	4′-hydroxy-3,3′,4,5,5′-pentamethoxy-7,9′:7′,9-diepoxylignane (**33**)	14 μM TE				

	Liriodendrin (**34**)	2.5 μM TE			
	Pinoresinol-4′-O-β-D-glucopyranoside (**35**)	8.5 μM TE			
	Blumenol-*C*-glucoside (**46**)	5 μM TE			
	Citrusin C (**39**)	2 μM TE			
	Coniferin (**40**)	6 μM TE			
	(*Z*)-plicatin B (**44**)	14.5 μM TE			
	Glycinol-3-*O*-β-*D*-glucopyranoside (**41**)	0 μM TE	CUPRAC	nd	nd
	Ismaeloside A (**25**)	1.5 μM TE	(1 μM)		
	Daidzin (**20**)	0.1 μM TE			
	Daidzein-4′-*O*-β-*D*-glucopyranoside (**22**)	0 μM TE			
	Genistin (**21**)	0.5 μM TE			
	Prunetrin (**23**)	0.1 μM TE			
	Coumestrol (**42**)	1 μM TE			
	Isobavachalcone (**24**)	0.9 μM TE			
	4′-hydroxy-3,3′,4,5,5′-pentamethoxy-7,9′:7′,9-diepoxylignane (**33**)	2 μM TE			
	Liriodendrin (**34**)	0.1 μM TE			
	Pinoresinol-4′-O-β-D-glucopyranoside (**35**)	0.1 μM TE			
	Blumenol-*C*-glucoside (**46**)	0.1 μM TE			
	Citrusin C (**39**)	0 μM TE			
	Coniferin (**40**)	0.1 μM TE			
	(*Z*)-plicatin B (**44**)	0.7 μM TE			
			CUPRAC		
	Glycinol-3-*O*-β-*D*-glucopyranoside (**41**)	1 μM TE	(10 μM)		
	Ismaeloside A (**25**)	6 μM TE			
	Daidzin (**20**)	2 μM TE			

	Daidzein-4′-*O*-β-*D*-glucopyranoside (**22**)	1.7 μM TE				
	Genistin (**21**)	2.5 μM TE				
	Prunetrin (**23**)	1.6 μM TE				
	Coumestrol (**42**)	6.3 μM TE				
	Isobavachalcone (**24**)	2 μM TE				
	4′-hydroxy-3,3′,4,5,5′-pentamethoxy-7,9′:7′,9-diepoxylignane (**33**)	24 μM TE				
	Liriodendrin (**34**)	0.1 μM TE				
	Pinoresinol-4′-O-β-D-glucopyranoside (**35**)	6 μM TE				
	Blumenol-*C*-glucoside (**46**)	0.7 μM TE				
	Citrusin C (**39**)	0 μM TE				
	Coniferin (**40**)	0.2 μM TE				
	(*Z*)-plicatin B (**44**)	4.7 μM TE				
	EO of AP (1000 μg/mL)	TAC: 68.5 ± 2.2%	BCBA	Rutin (1000 μg/mL)	TAC: 91.2 ± 2.9%	[42]
	EO of AP (10 μg/mL)	I: 17.9 ± 1.8%	DPPH	Ascorbic acid (10 μg/mL)	I: 71.8 ± 2.1%	
	EO of AP (50 μg/mL)	I: 28.8 ± 3.0%		Ascorbic acid (50 μg/mL)	I: 80.2 ± 3.1%	
	EO of AP (100 μg/mL)	I: 40.2± 2.5%		Ascorbic acid (100 μg/mL)	I: 87.5 ± 2.4%	
	EO of AP (500 μg/mL)	I: 66.9 ± 1.9%		Ascorbic acid (500 μg/mL)	I: 92.2 ± 3.0%	
	EO of AP (1000 μg/mL)	I: 72.1 ± 2.8%		Ascorbic acid (1000 μg/mL)	I: 94.2 ± 2.8%	

Antiproliferative and cytotoxic	*D. anethifolia*	Pabulenol (**16**)	ED_{50}: 30.47 ± 0.47 μM	MP	Doxorubicin	AA: 0.054 ± 0.005 μM	PAR cell line	[2]
		(+)-Oxypeucedanin hydrate (**14**)	ED_{50}: 41.96 ± 0.88 μM					
		Oxypeucedanin (**3**)	ED_{50}: 25.98 ± 1.27 μM					
		Oxypeucedanin methanolate (**4**)	ED_{50}: 35.88 ± 0.96 μM					
		Imperatorin (**12**)	ED_{50}: 36.12 ± 0.91 μM					
		Isogospherol (**6**)	ED_{50}: 46.53 ± 0.47 μM					
		Heraclenin (**9**)	ED_{50}: 32.73 ± 2.40 μM					
		Heraclenol (**11**)	ED_{50}: 52.31 ± 2.12 μM					
		Pabulenol (**16**)	ED_{50}: 29.28 ± 0.45 μM		Doxorubicin	AA: 0.468 ± 0.065 μM	MDR cell line	
		(+)-Oxypeucedanin hydrate (**14**)	ED_{50}: 60.58 ± 2.74 μM					
		Oxypeucedanin (**3**)	ED_{50}: 28.89 ± 0.73 μM					
		Oxypeucedanin methanolate (**4**)	ED_{50}: 33.23 ± 0.51 μM					
		Imperatorin (**12**)	ED_{50}: 42.24 ± 0.88 μM					
		Isogospherol (**6**)	ED_{50}: 48.75 ± 0.28 μM					
		Heraclenin (**9**)	ED_{50}: 46.54 ± 1.22 μM					
		Heraclenol (**11**)	ED_{50}: 46.57 ± 0.47 μM					
		Pabulenol (**16**)	ED_{50}: 51.32 ± 3.32 μM		Doxorubicin	CA: 0.377 ± 0.02 μM	PAR cell line	
		(+)-Oxypeucedanin hydrate (**14**)	ED_{50}: >100 μM					
		Oxypeucedanin (**3**)	ED_{50}: 40.33 ± 0.63 μM					
		Oxypeucedanin methanolate (**4**)	ED_{50}: 56.42 ± 5.23 μM					
		Imperatorin (**12**)	ED_{50}: 52.56 ± 4.19 μM					
		Isogospherol (**6**)	ED_{50}: >100 μM					
		Heraclenin (**9**)	ED_{50}: 65.81 ± 1.00 μM					
		Heraclenol (**11**)	ED_{50}: >100 μM					

	Pabulenol (**16**)	ED_{50}: >100 μM		Doxorubicin	CA: 7.152 ± 0.358 μM	MDR cell line
	(+)-Oxypeucedanin hydrate (**14**)	ED_{50}: >100 μM				
	Oxypeucedanin (**3**)	ED_{50}: 66.68 ± 0.00 μM				
	Oxypeucedanin methanolate (**4**)	ED_{50}: >100 μM				
	Imperatorin (**12**)	ED_{50}: >100 μM				
	Isogospherol (**6**)	ED_{50}: >100 μM				
	Heraclenin (**9**)	ED_{50}: 83.94 ± 1.68 μM				
	Heraclenol (**11**)	ED_{50}: >100 μM				
	Pabulenol (**16**)	ED_{50}: 54.09 ± 3.83 μM		Doxorubicin	CA: 5.71 ± 0.50 μM	NIH/3T3 (ATCC-CRL-1658) cell line
	(+)-Oxypeucedanin hydrate (**14**)	ED_{50}: 83.55 ± 0.57 μM				
	Oxypeucedanin (**3**)	ED_{50}: 57.18 ± 3.91 μM				
	Oxypeucedanin methanolate (**4**)	ED_{50}: 47.16 ± 1.28 μM				
	Imperatorin (**12**)	ED_{50}: 92.41 ± 2.80 μM				
	Isogospherol (**6**)	ED_{50}: 54.82 ± 1.99 μM				
	Heraclenin (**9**)	ED_{50}: 70.91 ± 4.26 μM				
	Heraclenol (**11**)	ED_{50}: 65.78 ± 0.46 μM				
	Oxypeucedanin (**3**) + doxorubicin (1:50)	ED_{50}: 0.85 ± 0.07 μM	CCA			
	Heraclenin (**9**) + doxorubicin (4:100)	ED_{50}: 0.88 ± 0.06 μM				

		Ducrosin A (**26**)	IC_{50}: 56.0 ± 8.0 μM	MTT	Tamoxifen	IC_{50}: 1.0 ± 0.2 μM	HCT-116 cell line	[53]
		Ducrosin B (**28**)	IC_{50}: 41.0 ± 3.0 μM					
		Stigmasterol (**36**)	IC_{50}: >100 μM					
		Heraclenin (**9**)	IC_{50}: 52.5 ± 5.0 μM					
		Heraclenol (**11**)	IC_{50}: >100 μM					
		Ducrosin A (**26**)	IC_{50}: 89.0 ± 11.0 μM		Tamoxifen	IC_{50}: 1.4 ± 0.3 μM	SKOV-3 cell line	
		Ducrosin B (**28**)	IC_{50}: 54.0 ± 3.0 μM					
		Stigmasterol (**36**)	IC_{50}: >100 μM					
		Heraclenin (**9**)	IC_{50}: 60.0 ± 5.0 μM					
		Heraclenol (**11**)	IC_{50}: 97.0 ± 13.0 μM					
		EO of AP	IC_{50}: 85.5 ± 29.2 μg/mL	MTT	Cisplatin	IC_{50}: 6.9 ± 3.6 μg/mL	K562 cell line	[8]
			IC_{50}: 197.8 ± 38.2 μg/mL			IC_{50}: 3.5 ± 0.8 μg/mL	LS180 cell line	
			IC_{50}: 321.4 ± 88.7 μg/mL			IC_{50}: 5.0 ± 1.5 μg/mL	MCF-7 cell line	
	D. flabellifolia	EO of AP	IC_{50}: 304.0 ± 87.2 μg/mL	MTT	Cisplatin	IC_{50}: 6.9 ± 3.6 μg/mL	K562 cell line	[8]
			IC_{50}: 286.9 ± 28.0 μg/mL			IC_{50}: 3.5 ± 0.8 μg/mL	LS180 cell line	
			IC_{50}: 511.2 ± 133.2 μg/mL			IC_{50}: 5.0 ± 1.5 μg/mL	MCF-7 cell line	

		EtOH (95%) Ex.	IC_{50}: 25.34 ± 0.68 μg/mL	MTT	Vincristine sulphate	IC_{50}: 11.02 ± 0.05 μg/mL	MCF-7 cell line	[70]
		Ch (95%) Ex.	IC_{50}: 47.25 ± 1.75 μg/mL					
		Aqueous Ex.	IC_{50}: 33.04 ± 1.55 μg/mL					
		EtOH (95%) Ex.	IC_{50}: 98.01 ± 1.80 μg/mL		Vincristine sulphate	IC_{50}: > 90 μg/mL	HEp-2 cell line	
		Ch (95%) Ex.	IC_{50}: 144.35 ± 0.97 μg/mL					
		Aqueous Ex.	IC_{50}: 159.33 ± 1.36 μg/mL					
		EtOH (95%) Ex.	IC_{50}: 87.50 ± 1.65 μg/mL		Vincristine sulphate	IC_{50}: > 90 μg/mL	Vero cell line	
		Ch (95%) Ex.	IC_{50}: 152.33 ± 1.98 μg/mL					
		Aqueous Ex.	IC_{50}: 164.06 ± 0.88 μg/mL					
	D. ismaelis	EO of AP	IC_{50}: 66.24 ± 1.26 μg/mL	MTT	Vinblastine	IC_{50}: 1.8 ± 0.26 μg/mL	MCF-7 cell line	[42]
			IC_{50}: 137.32 ± 2.48 μg/mL			IC_{50}: 2.5 ± 0.38 μg/mL	HEpG2 cell line	
			IC_{50}: 102.53 ± 1.0 μg/mL			IC_{50}: 2.4 ± 0.8 μg/mL	LoVo cell line	
Enzyme inhibition	*D. anethifolia*	MeOH Ex. (80% v/v) of AP (1 μg/mL)	NQO1 SA: 1 t/c	NQO1S	Sulforaphane (0.1 μg/mL)	NQO1 SA: 1.4 t/c	Murine hepatoma (Hepa1c1c7) cell line	[67]
		MeOH Ex. (80% v/v) of AP (10 μg/mL)	NQO1 SA: 1.3 t/c		Sulforaphane (0.5 μg/mL)	NQO1 SA: 2.5 t/c		
		MeOH Ex. (80% v/v) of AP (100 μg/mL)	NQO1 SA: 2.5 t/c		Sulforaphane (1 μg/mL)	NQO1 SA: 4 t/c		
					Sulforaphane (5 μg/mL)	NQO1 SA: 4.8 t/c		
		MeOH Ex. (80% v/v) of AP	CD: 32 μg/mL		Sulforaphane (0.04-2.5 μM)	CD: 35.45 μg/mL		
		MeOH Ex. (80% v/v) of AP	MMI: 2.5-fold		Sulforaphane (nd)	MMI: 4.7-fold		
Hepatotoxicity	*D. anethifolia*	EO of L and St	ALP: 3.61 ± 0.06 μmol/mL		Normal control	ALP: 3.46 ± 0.08 μmol/mL		[51]
		STZ + EO of L and St	ALP: 5.00 ± 0.13 μmol/mL		STZ + Glibenclamide	ALP: 4.03 ± 0.38 μmol/mL		

Hypoglycaemic	*D. anethifolia*	Ex. of L and St	TC: 53.03 ± 5.45 ug/dL	Normal control	TC: 49.60 ± 10.95 ug/dL	Albino rats (*in vivo*)	[51]
		STZ + Ex. of L and St	TC: 87.00 ± 9.23 ug/dL	STZ + Glibenclamide	TC: 84.69 ± 8.24 ug/dL		
		Ex. of L and St	TG: 25.20 ± 2.00 ug/dL	Normal control	TG: 24.72 ± 7.16 ug/dL		
		STZ + Ex. of L and St	TG: 39.40 ± 1.90 ug/dL	STZ + Glibenclamide	TG: 38.18 ± 1.57 ug/dL		
		Ex. of L and St	HDL: 31.29 ± 3.16 mg/dL	Normal control	HDL: 30.21 ± 2.48 mg/dL		
		STZ + Ex. of L and St	HDL: 18.23 ± 1.09 mg/dL	STZ + Glibenclamide	HDL: 18.82 ± 1.62 mg/dL		
		Ex. of L and St	LDL: 13.00 ± 1.09 ug/dL	Normal control	LDL: 12.49 ± 9.24 ug/dL		
		STZ + Ex. of L and St	LDL: 39.96 ± 11.00 ug/dL	STZ + Glibenclamide	LDL: 38.23 ± 4.65 ug/dL		
		Ex. of L and St	TL: 1105.26 ± 52.65 ug/dL	Normal control	TL: 1100.00 ± 52.6 ug/dL		
		STZ + Ex. of L and St	TL: 1510.66 ± 60.20 ug/dL	STZ + Glibenclamide	TL: 1421.00 ± 59.60 ug/dL		

Immunostimulator y	*D. anethifolia*	EO of AP (0 Day)	SBA: 1.04 ± 0.38 col/day	nd	PBS (0 Day)	SBA: 0.58 ± 0.76 C/day	Fish (*in vivo*)	[66]
		EO of AP (10 Day)	SBA: 0.85 ± 0.38 col/day		PBS (10 Day)	SBA: 1.41 ± 0.76 C/day		
		EO of AP (20 Day)	SBA: 3.83 ± 0.38 col/day		PBS (20 Day)	SBA: 8.75 ± 0.76 C/day		
		EO of AP (30 Day)	SBA: 1.08 ± 0.38 col/day		PBS (30 Day)	SBA: 0.50 ± 0.76 C/day		
		EO of AP (40 Day)	SBA: 0.72 ± 0.38 col/day		PBS (40 Day)	SBA: 0.75 ± 0.76 C/day		
		EO of AP (0 Day)	SLA: 1583.33 ± 447.19 u/mL	nd	PBS (0 Day)	SLA: 1416 ± 894 U/mL		
		EO of AP (10 Day)	SLA: 1416.67 ± 447.19 u/mL		PBS (10 Day)	SLA: 1375 ± 894 U/mL		
		EO of AP (20 Day)	SLA: 2562.50 ± 447.19 u/mL		PBS (20 Day)	SLA: 4333 ± 894 U/mL		
		EO of AP (30 Day)	SLA: 1687.50 ± 447.19 u/mL		PBS (30 Day)	SLA: 2291 ± 894 U/mL		
		EO of AP (40 Day)	SLA: 3239.58 ± 447.19 u/mL		PBS (40 Day)	SLA: 2625 ± 894 U/mL		
		EO of AP (0 Day)	RBA: 0.968 ± 0.029 OD	nd	PBS (0 Day)	RBA: 0.928 ± 0.05 OD		
		EO of AP (10 Day)	RBA: 0.857 ± 0.027 OD		PBS (10 Day)	RBA: 0.767 ± 0.05 OD		
		EO of AP (20 Day)	RBA: 0.820 ± 0.027 OD		PBS (20 Day)	RBA: 0.777 ± 0.05 OD		
		EO of AP (30 Day)	RBA: 0.697 ± 0.027 OD		PBS (30 Day)	RBA: 0.619 ± 0.05 OD		
		EO of AP (40 Day)	RBA: 0.954 ± 0.027 OD		PBS (40 Day)	RBA: 0.923 ± 0.05OD		
		EO of AP (0.001%)	SBA: 0.90 ± 0.34 col/day	nd	PBS (0.04 M)	SBA: 2.40 ± 0.34 C/day		
		EO of AP (0.01%)	SBA: 1.31 ± 0.34 col/day					
		EO of AP (0.1%)	SBA: 1.41 ± 0.34 col/day					
		EO of AP (0.001%)	SLA: 1883.33 ± 366.05 u/mL	nd	PBS (0.04 M)	SLA: 2408.33 ± 366.05 U/mL		
		EO of AP (0.01%)	SLA: 1950 ± 366.05 u/mL					
		EO of AP (0.1%)	SLA: 150 ± 366.05 u/mL					
		EO of AP (0.001%)	RBA: 0.776 ± 0.030 OD	nd	PBS (0.04 M)	RBA: 0.803 ± 0.029 OD		

	EO of AP (0.01%)	RBA: 0.957 ± 0.029 OD			
	EO of AP (0.1%)	RBA: 0.901 ± 0.029 OD			
	EO of AP (0.001% - 0 Day)	SBA: 0.58 ± 0.76 col/day	nd	nd	nd
	EO of AP (0.001% - 10 Day)	SBA: 1.41 ± 0.76 col/day			
	EO of AP (0.001% - 20 Day)	SBA: 8.75 ± 0.76 col/day			
	EO of AP (0.001% - 30 Day)	SBA: 0.50 ± 0.76 col/day			
	EO of AP (0.001% - 40 Day)	SBA: 0.75 ± 0.76 col/day			
	EO of AP (0.001% - 0 Day)	SLA: 1541 ± 894 u/mL	nd	nd	nd
	EO of AP (0.001% - 10 Day)	SLA: 1416 ± 894 u/mL			
	EO of AP (0.001% - 20 Day)	SLA: 2750 ± 894 u/mL			
	EO of AP (0.001% - 30 Day)	SLA: 1125 ± 894 u/mL			
	EO of AP (0.001% - 40 Day)	SLA: 2583 ± 894 u/mL			
	EO of AP (0.001% - 0 Day)	RBA: 894 1.019 ± 0.06 OD	nd	nd	nd
	EO of AP (0.001% - 10 Day)	RBA: 894 0.738 ± 0.05 OD			
	EO of AP (0.001% - 20 Day)	SIA: 894 0.757 ± 0.05 OD			
	EO of AP (0.001% - 30 Day)	SIA: 894 0.596 ± 0.05 OD			
	EO of AP (0.001% - 40 Day)	SIA: 894 0.771 ± 0.05 OD			
	EO of AP (0.01% - 0 Day)	SBA: 2.00 ± 0.76 col/day	nd	nd	nd
	EO of AP (0.01% - 10 Day)	SBA: 0.75 ± 0.76 col/day			
	EO of AP (0.01% - 20 Day)	SBA: 1.25 ± 0.76 col/day			
	EO of AP (0.01% - 30 Day)	SBA: 1.91 ± 0.76 col/day			
	EO of AP (0.01% - 40 Day)	SBA: 0.66 ± 0.76 col/day			

	EO of AP (0.01% - 0 Day)	SLA: 1791 ± 894 u/mL	nd	nd	nd
	EO of AP (0.01% - 10 Day)	SLA: 958 ± 894 u/mL			
	EO of AP (0.01% - 20 Day)	SLA: 1416 ± 894 u/mL			
	EO of AP (0.01% - 30 Day)	SLA: 1500 ± 894 u/mL			
	EO of AP (0.01% - 40 Day)	SLA: 4083 ± 894 u/mL			
	EO of AP (0.01% - 0 Day)	RBA: 1.01 ± 0.06 OD	nd	nd	nd
	EO of AP (0.01% - 10 Day)	RBA: 0.73 ± 0.05 OD			
	EO of AP (0.01% - 20 Day)	RBA: 0.75 ± 0.05 OD			
	EO of AP (0.01% - 30 Day)	RBA: 0.59 ± 0.05 OD			
	EO of AP (0.01% - 40 Day)	RBA: 0.77 ± 0.05 OD			
	EO of AP (0.1% - 0 Day)	SBA: 0.66 ± 0.76 col/day	nd	nd	
	EO of AP (0.1% - 10 Day)	SBA: 0.41 ± 0.76 col/day			
	EO of AP (0.1% - 20 Day)	SBA: 4.58 ± 0.76 col/day			
	EO of AP (0.1% - 30 Day)	SBA: 0.66 ± 0.76 col/day			
	EO of AP (0.1% - 40 Day)	SBA: 0.75 ± 0.76 col/day			
	EO of AP (0.1% - 0 Day)	SLA: 1583 ± 894 u/mL	nd	nd	
	EO of AP (0.1% - 10 Day)	SLA: 1916 ± 894 u/mL			
	EO of AP (0.1% - 20 Day)	SLA: 1750 ± 894 u/mL			
	EO of AP (0.1% - 30 Day)	SLA: 1833 ± 894 u/mL			
	EO of AP (0.1% - 40 Day)	SLA: 3666 ± 894 u/mL			
	EO of AP (0.1% - 0 Day)	RBA: 0.887 ± 0.05 OD	nd	nd	
	EO of AP (0.1% - 10 Day)	RBA: 0.967 ± 0.05 OD			
	EO of AP (0.1% - 20 Day)	RBA: 0710 ± 0.05 OD			

	EO of AP (0.1% - 30 Day)	RBA: 0.892 ± 0.05 OD
	EO of AP (0.1% - 40 Day)	RBA: 1.047 ± 0.05 OD

Neuroprotective	*D. anethifolia*	EO of AP (0.5 mL/kg w)	AT-4: 31 s on 1st day	MWM	Saline (0.5 mL)	ATRP: 39 s on 1st day	Rat (*in vivo*)	[65]
			AT-4: 24 s on 2nd day		Saline (0.5 mL)	ATRP: 32 s on 2nd day		
			AT-4: 20 s on 3rd day		Saline (0.5 mL)	ATRP: 28 s on 3rd day		
			AT-4: 12 s on 4th day		Saline (0.5 mL)	ATRP: 28 s on 4th day		
			AD: 700 cm on 1st day		Saline (0.5 mL)	ADTRP: 800 cm on 1st day		
			AD:520 cm on 2nd day		Saline (0.5 mL)	ADTRP: 650 cm on 2nd day		
			AD: 420 cm on 3rd day		Saline (0.5 mL)	ADTRP: 640cm on 3rd day		
			AD: 310 cm on fourth day		Saline (0.5 mL)	ADTRP: 655 cm on 4th day		
			AS: 52 cm/s on 1st day		Saline (0.5 mL)	ASRP: 69 cm/s on 1st day		
			AS: 57 cm/s on 2nd day		Saline (0.5 mL)	ASRP: 67cm/s on 2nd day		
			AS: 59 cm/s on 3rd day		Saline (0.5 mL)	ASRP: 65 cm/s on 3rd day		
			AS: 59 cm/s on 4th day		Saline (0.5 mL)	ASRP: 60 cm/s on 4th day		
			TD: 38%		Saline (0.5 mL)	DTQT: 31%		
			TD: 30%		Saline (0.5 mL)	ATQT: 32 s		
			TD: 33%		Saline (0.5 mL)	TPZ: 58%		
		EO of AP (0.25 mL/kg w)	AT-4: 34 s on 1st day					
			AT-4: 24 s on 2nd day					
			AT-4: 23 s on 3rd day					
			AT-4: 14 s on 4th day					
			AD: 790 cm on 1st day					
			AD: 500 cm on 2nd day					
			AD: 480 cm on 3rd day					

	AD: 440 cm on 4th day
	AS: 57 cm/s on 1st day
	AS: 55 cm/s on 2nd day
	AS: 62 cm/s on 3rd day
	AS: 58 cm/s on 4th day
	AT-5: 41 s
	AT-5: 36 s
	AT-5: 31 s
EO of AP (0.125 mL/kg w)	AT-4: 34 s on 1st day
	AT-4: 27 s on 2nd day
	AT-4: 18 s on 3rd day
	AT-4: 22 s on 4th day
	AD: 630 cm on 1st day
	AD: 490 cm on 2nd day
	AD: 560 cm on 3rd day
	AD: 550 cm on 4th day
	AS: 58 cm/s on 1st day
	AS: 56 cm/s on 2nd day
	AS: 64 cm/s on 3rd day
	AS: 59 cm/s on 4th day
	TPZ: 33%

			TPZ: 49%<br>TPZ: 45%					
		Control (no EO)	ATRP: 37 s on 1st day<br>ATRP: 29 s on 2nd day<br>ATRP: 30 s on 3rd day<br>ATRP: 28 s on 4th day					
			ADTRP: 830 s on 1st day<br>ATRP: 700 s on 2nd day<br>ATRP: 680 s on 3rd day<br>ATRP: 720 s on 4th day					
			ASRP: 59 s on 1st day<br>ATRP: 58 s on 2nd day<br>ATRP: 62 s on 3rd day<br>ATRP: 64 s on 4th day					
			DTQT: 30%<br>ATQT: 32 s<br>TPZ: 54%					
Testosterone hormone	*D. anethifolia*	EtOH Ex. AP (80%, 140 mg/kg w)<br>EtOH Ex. AP (80%, 280 mg/kg w)<br>EtOH Ex. AP (80%, 560 mg/kg w)	TO: 0.24 ng/mL<br>TO: 1 ng/mL<br>TO: 1.25 ng/mL	RM	control group	TO: 2.4 ng/mL	Wistar rats/*in vivo*	[68]

AA: antiproliferative activity; AcP: Acute phase; AD: average distance; AI: α-amylase inhibition; AP: aerial part; ALT: alanine aminotransferase; ALP: alkaline phosphatase; AS: average speed; AST: aspartate aminotransferase; AT-4: average time on 4th day; AT-5: average time on 5th day; BCBA: β-carotene bleaching assay; BGL: blood glucose level; BHA: butylated hydroxy anisole; BHT: butylated hydroxytoluene; CA: cytotoxic activity; CCA: checkboard combination assay; CD: concentration which doubles the specific activity of NQO1; CFU: colony-forming unit; CFUC: colony-forming unit per gram counting; Ch: chloroform; CMEI: carbohydrates metabolizing enzymes inhibition assay; col: colonies; CP: chronic phase; Cr: creatinine; CUPRAC: copper reducing capacity; DCM: dichloromethane; DD: disk diffusion; DLTCS: dose-dependent length of tonic-clonic seizure; DLTS: dose-dependent length of tonic seizure; DMS: duration of myoclonic seizure; DPPH: 2,2-diphenyl-1-picrylhydrazyl; DR: DPPH remaining; DRTJ: delayed response of mouse tail jump; DTCS: duration of tonic-clonic seizure; DTS: duration of tonic seizure; EC_{50}: half maximal effective concentration; ED_{50}: median effective dose; EO: essential oil; EPM: elevated plus maze; EtOAc: ethyl acetate; EtOH: ethanol; ES: ear swelling; Ex.: extract; Fl: flowering stage; Fr: fruiting stage; FRAP: ferric reducing antioxidant of potency; GI: α-glucosidase inhibition activity; GIA: β-galactosidase inhibitory activity; HCT: human colon cancer cell line; HDL: high-density lipoprotein cholesterol; HEp-2: Larynx carcinoma cell line; HEpG2: liver cancer cell line; HK: hexokinase; HPS: hydrogen peroxide scavenging; I: inhibition; IC_{50}: half maximal inhibitory concentration; IZ: inhibition zone; K562: human chronic myelogenous leukemia cell line; L: leaf; LA: locomotor; LAA: locomotor activity apparatus; LDH: lactate dehydrogenase; LDL: low-density lipoprotein cholesterol; LoVo: colon cancer cell line; LS: latency to sleep; LS180: human colon adenocarcinoma cell line; LTBSA: lengthened the time interval before the beginning of the seizure attacks; M: mortality; MBC: minimum bactericidal concentration; MbD: micro broth dilution; MCF-7: human breast adenocarcinoma cell line; MDR: multidrug resistant cells; MeOH: methanol; MFC: minimum fungicidal concentration; MIC: minimum inhibitory concentration; MMI: maximum magnitude of induction; MP: microtiter plate assay; MR: methicillin resistant; MS: methicillin sensitive; MWM: Morris Water Maze assay; nd: not determined; NIH/3T3: normal murine fibroblast cell; no: number; NORS: nitric oxide radical scavenging; NQO1S: NAD(P)H:quinone acceptor oxidoreductase 1 specific assay; NQO1 SA: NAD(P)H:quinone acceptor oxidoreductase 1 specific activity; OAE: open arm entry; OAT: open arm time; OD: optical density; ORAC: oxygen radical absorbance capacity; P: protection; PAR: mouse T-lymphoma parental cell line; PBS: phosphate-buffered saline; PEPCK: phosphoenolpyruvate carboxy kinase; PK: pyruvate kinase; PTZ: pentylenetetrazole; RBA: respiratory burst activity; RM: radioimmunoassay; RS: record of seizure-related traits; s: second; SARS: superoxide anion radical scavenging; SBA: serum bactericidal activity; Sc: score; SKOV-3: ovarian carcinoma cancer cell line; SLA: serum lysozyme activity; St: stem; ST: sleeping time; STZ: streptozotocin; t/c: treated/control; TAC: total antioxidant capacity; TC: total cholesterol; TD: travelled distance; TDSL: total dose-dependent seizure length; TE: Trolox equivalent; TFC: total fungi counts; TG: triglycerides; TL: total lipids; TO: testosterone concentration; TPZ: time in peripheral zones; TRAP: tartrate-resistant acid phosphatase; TU: total urea; u: unit; Vero: African green monkey kidney cell line
